# An integrated nationwide genomics study reveals transmission modes of typhoid fever in China

**DOI:** 10.1128/mbio.01333-23

**Published:** 2023-10-06

**Authors:** Ye Feng, Hang Pan, Beiwen Zheng, Fang Li, Lin Teng, Zhijie Jiang, Mengyao Feng, Xiao Zhou, Xianqi Peng, Xuebin Xu, Haoqiu Wang, Beibei Wu, Yonghong Xiao, Stephen Baker, Guoping Zhao, Min Yue

**Affiliations:** 1 Sir Run Run Shaw Hospital, Zhejiang University School of Medicine, Hangzhou, China; 2 Institute of Translational Medicine, Zhejiang University School of Medicine, Hangzhou, China; 3 Department of Veterinary Medicine, Zhejiang University College of Animal Sciences, Hangzhou, China; 4 State Key Laboratory for Diagnosis and Treatment of Infectious Diseases, National Clinical Research Center for Infectious Diseases, National Medical Center for Infectious Diseases, The First Affiliated Hospital, Zhejiang University School of Medicine, Hangzhou, China; 5 Shanghai Municipal Center for Disease Control and Prevention, Shanghai, China; 6 Hangzhou Center for Disease Control and Prevention, Hangzhou, China; 7 Zhejiang Province Center for Disease Control and Prevention, Hangzhou, China; 8 School of Public Health and Managemet, Wenzhou Medical University, Wenzhou, Zhejiang, China; 9 University of Cambridge School of Clinical Medicine, Cambridge Biomedical Campus, Cambridge, United Kingdom; 10 School of Life Science, Hangzhou Institute for Advanced Study, University of Chinese Academy of Sciences, Hangzhou, China; 11 CAS Key Laboratory of Synthetic Biology, Institute of Plant Physiology and Ecology, Shanghai Institutes for Biological Sciences, Chinese Academy of Sciences, Shanghai, China; 12 Department of Microbiology and Microbial Engineering, School of Life Sciences, Fudan University, Shanghai, China; 13 Hainan Institute of Zhejiang University, Sanya, China; University of Cambridge, Cambridge, United Kingdom

**Keywords:** *Salmonella *Typhi, typhoidal fever, transmission, exploiting genomics, pandemic clone

## Abstract

Typhoid fever, caused by *Salmonella* Typhi (*S*. Typhi), is a life-threatening disease, usually food-borne and commonly associated with international travel. The disease transmission remains endemic in many low- and middle-income countries, representing further hotspots for seeding new global outbreaks. China has historically been affected by typhoid fever, but the respective roles of local transmission and importation remain unknown. Here, we generated a nationwide map of the typhoid burden in China and investigated the associations between typhoid disease, climate and various socioeconomic parameters. To assess transmission dynamics, we sub-sampled *S*. Typhi isolated within China over five decades and sequenced their genomes. The resulting 705 new genomes, placed in context with 5,190 global isolates from 87 countries on six continents, led to the discovery of several predominant inland Chinese clones belonging to the clades 2.1/2.3/3.2/4.3. These clones were associated with multiple introductions from overseas, followed by local expansion. Notably, 4.3.1 isolates from eastern China were not genetically close to those from northwestern China but to the international isolates, indicating their association with international travel. Additional *in vitro* assays showed that 4.3.1 elaborated better intracellular survival, acid tolerance, and desiccation tolerance than other lineages, partially explaining its success. For the first time, we have probed typhoid transmission in China, finding local transmission and importation, which could guide the policy for typhoid control.

**IMPORTANCE:**

Typhoid fever is a life-threatening disease caused by *Salmonella enterica* serovar Typhi, resulting in a significant disease burden across developing countries. Historically, China was very much close to the global epicenter of typhoid, but the role of typhoid transmission within China and among epicenter remains overlooked in previous investigations. By using newly produced genomics on a national scale, we clarify the complex local and global transmission history of such a notorious disease agent in China spanning the most recent five decades, which largely undermines the global public health network.

## INTRODUCTION

*Salmonella enterica* serovar Typhi (*S*. Typhi) is a human-restricted bacterium that causes typhoid fever. The disease is estimated to cause ~21.7 million illnesses and 216,000 deaths annually (1). Typhoid transmission is known to occur through two modes: “long-cycle” transmission, occurs through long-term exposure to contamination in the broader environment (i.e., untreated water or sewage), whereas “short-cycle” transmission, refers to individuals exposed through close interaction with infected individuals via food handling and is often linked to travel (2). Typically, “long-cycle” transmission occurs in endemic countries with inadequate hygiene and safe food/water handling practices, including southern Asia, Africa, and South America (1, 3, 4), where the environment is the primary reservoir. In high-income countries with improved sanitation facilities, however, “short-cycle” transmission is dominant, with most cases associated with recent international travel (5), where humans are the main reservoir.

Genetically, *S*. Typhi was traditionally thought to be monophyletic, with limited nucleotide diversity and recombination capability, as revealed by multi-locus sequence typing technology (6 – 8). With the high resolution of whole-genome sequencing (WGS), the global *S*. Typhi population can be grouped into 5 primary clades and 24 subclades (9, 10). The clade 4.3.1, also termed H58, represents a common genotype that became widely distributed in southern Asia and the Middle East in the 1990s and later spread worldwide (11). Many of the 4.3.1 isolates are characterized by multi-drug resistance (MDR), including resistance against chloramphenicol, ampicillin, and trimethoprim-sulfamethoxazole. MDR was primarily driven by the acquisition of IncHI1 plasmids; however, the more recent integration of additional antimicrobial-resistance (AMR) genes and chromosomal point mutations (12, 13) has conferred resistance to fluoroquinolones, third-generation cephalosporins, and azithromycin in 4.3.1 (14 – 16).

China has vast geographical, meteorological, and economic diversity across its regions. Historically, typhoid fever was rampant across China. Over the past four decades, dramatic socioeconomic advancement has resulted in the overall incidence of typhoid in China dropping to low levels (17 – 19). Despite this reduction, the spatiotemporal heterogeneity of typhoid across China is unclear. Whether continuous population migration within China has generated heterogeneity in *S*. Typhi or disseminated endemic clones remains obscure. Additionally, transmission routes within China and potential importations have yet to be defined. To date, few large-scale studies have been conducted except for outbreak reports (20 – 22), and only 26 *S*. Typhi genome sequences from China are publicly available. Here, we amassed a nationwide collection of *S*. Typhi isolates spanning the last five decades and performed genome sequencing. In combination with global contextual genomes, we performed phylodynamic analysis and investigated the population structure and the scale of internal transmission and importation of *S*. Typhi in China.

## RESULTS

### Variable typhoid across China

**Fig 1:**
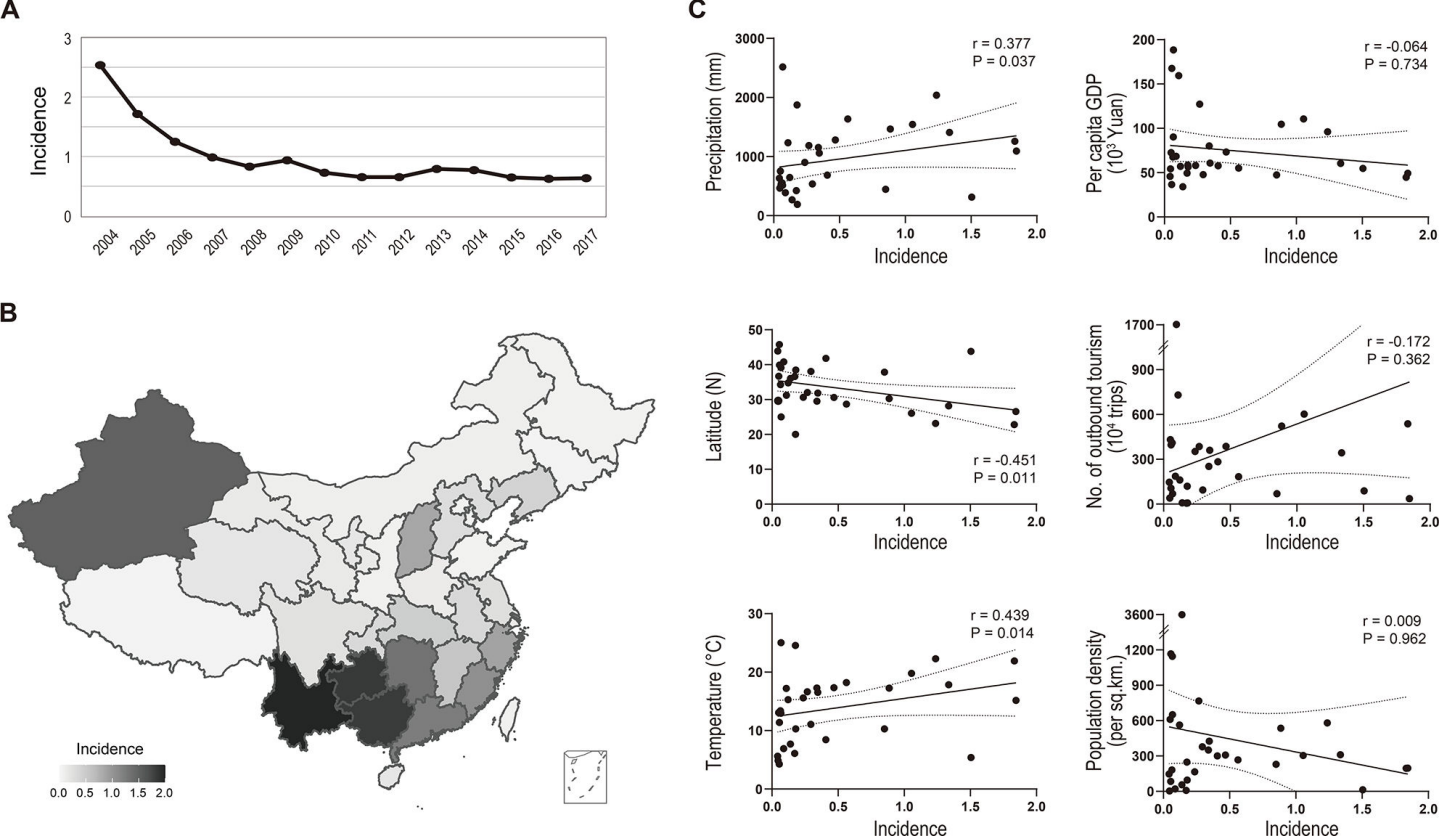
Incidence of typhoid fever in China. (**A**) Annual incidence (number of cases per 100,000 persons per year) of typhoid fever in China during 2004–2017. (**B**) The average incidence of typhoid fever in each province of China during 2008–2017. (**C**) Spearman correlation of climate, social, and economic factors (*y*-axis) with the incidence of typhoid fever (*x*-axis). Each dot indicates a province of China. The data for the plots are appended in Table S1.

The incidence of typhoid fever has continued to decline since 2004 and has remained constant at 0.8 cases per 100,000 persons per year since 2008 (Fig. 1A). The incidence varied between provinces and was generally found to be higher in northwestern and southern China (Fig. 1B). The incidence was correlated with various climatic factors such as temperature, precipitation, and latitude. However, incidence was not strongly associated with per capita gross domestic product (GDP), the number of outbound tourists, or population density (Fig. 1C; Table S1).

We collected the genomes of 731 *S*. Typhi isolates from 19 provinces across China. A global collection of 5,164 genomes retrieved from the public database was also included to provide a global context. The core genome of these isolates had a size of 3.97 Mb, within which a total of 42,702 core-genome single-nucleotide polymorphisms (cgSNPs) were identified. A Bayesian analysis of population structure typing and GenoTyphi, a 58 SNP-based genotyping scheme (9), were performed, respectively, and produced consistent typing results with each other (Fig. S1).

**Fig 2:**
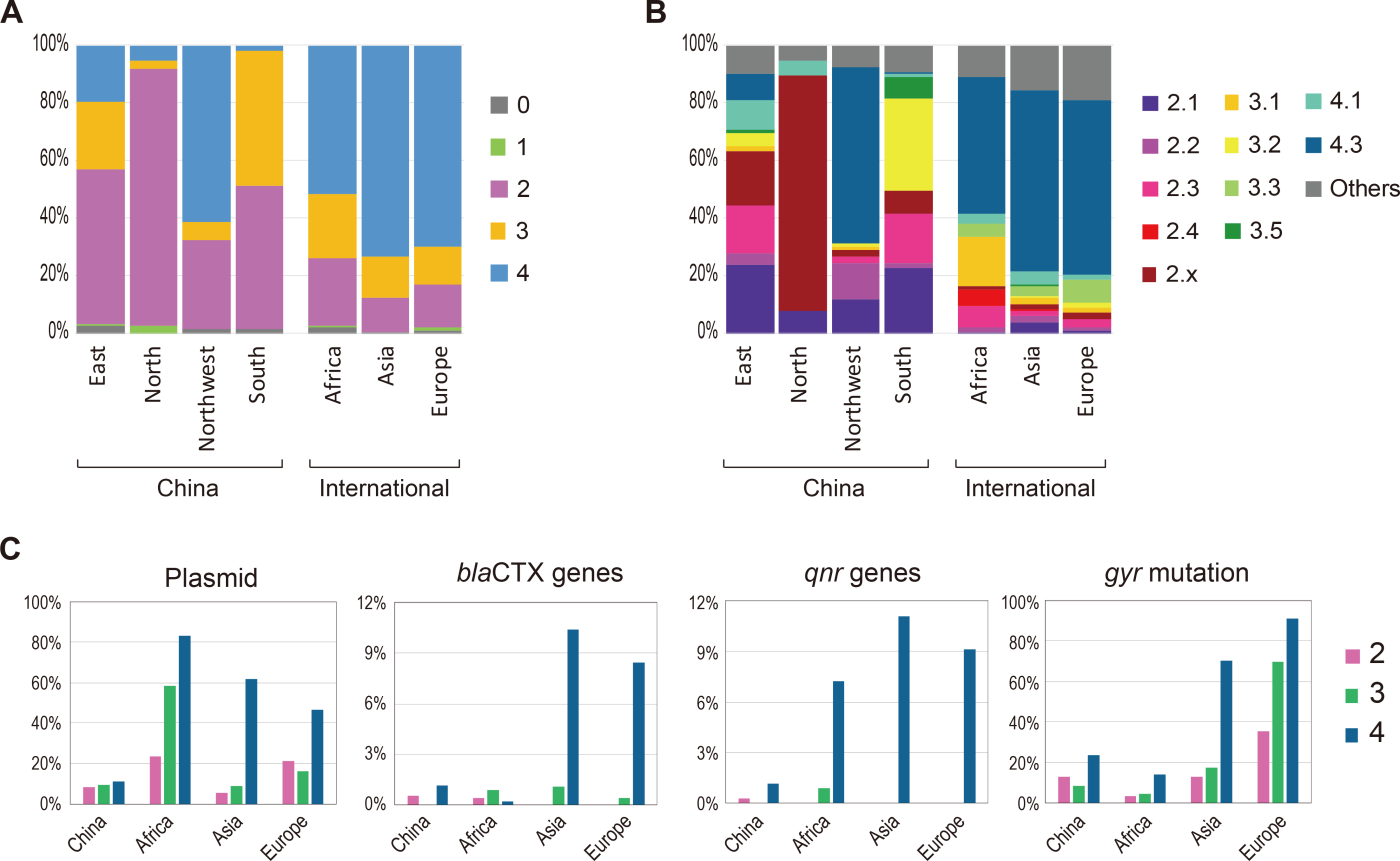
Distinct genotypic and antimicrobial patterns of *S*. Typhi between regions. The *S*. Typhi isolates are grouped into primary clades (**A**) and subclades (**B**) by the Genotyphi typing scheme. According to the geographic source, the comparison is made between different parts of China, i.e., eastern (*n* = 144), northern (*n* = 38), northwestern (*n* = 83), southern (*n* = 459), and between various regions over the world, i.e., China (*n* = 731), Africa (*n* = 1,074), Asia (excluding China, *n* = 1,443), and Europe (*n* = 1,942). (**C**) Comparison of carriage rate of antimicrobial-related genetic determinants.

The genotypic structure of the Chinese isolates was distinct between regions. Northern China was dominated by the primary clade 2, while other regions comprised two or three primary clades (Fig. 2A; Fig. S2). At the subclade level, 2.1 (20.8%), 2.3 (14.5%), 3.2 (21.2%), and 4.3 (9.0%) were dominant in China (Fig. 2B). No obvious clonal replacement was observed in any region during the last two decades (Fig. S3).

The demographic features of patients infected by 4.3 differed from those of other clades, with young people and females accounting for a higher proportion than the elders and males (Fig. S4). The division of the 4.3 patients according to the region further showed that the low age of the 4.3 patients was attributable to the patients from eastern China, and the sex bias was attributed to the patients from northwestern China.

International data were obtained from public databases. Overall, Africa, Asia, and Europe were dominated mainly by 4.3 (Fig. 2A and B), although the genotypic composition varied remarkably between and within continents (23).

### A low prevalence of AMR *S*. Typhi in China

We further analyzed the distribution of AMR-associated mobile genes and chromosomal mutations, particularly those responsible for resistance to front-line therapy. Only two Chinese *S*. Typhi isolates carried the plasmid-borne *qnr* genes for fluoroquinolone resistance. The *gyr* mutations were present in 12% of isolates and were mainly enriched in the primary clade 4, in which the occurrence was 24%. Three Chinese isolates, which belonged to different clades, carried distinct *bla*_CTX-M_ gene variants that offered resistance to third-generation cephalosporin. The recently emergent *acrB* mutations that mediate azithromycin resistance, as well as the *mcr*, *tetX*, and carbapenemase-encoding genes, which confer resistance to polymyxin, tigecycline and carbapenem, respectively, were not detected. The rate of plasmid carriage in Chinese isolates was <12% for all primary clades (Fig. 2C), with IncC and IncFIB (pHCM2) as the predominant replicon types (Fig. S5). By comparison, Asian and European isolates were much more resistant than Chinese isolates (Fig. 2C).

### Historic “long-cycle” transmission across provinces in China

**Fig 3:**
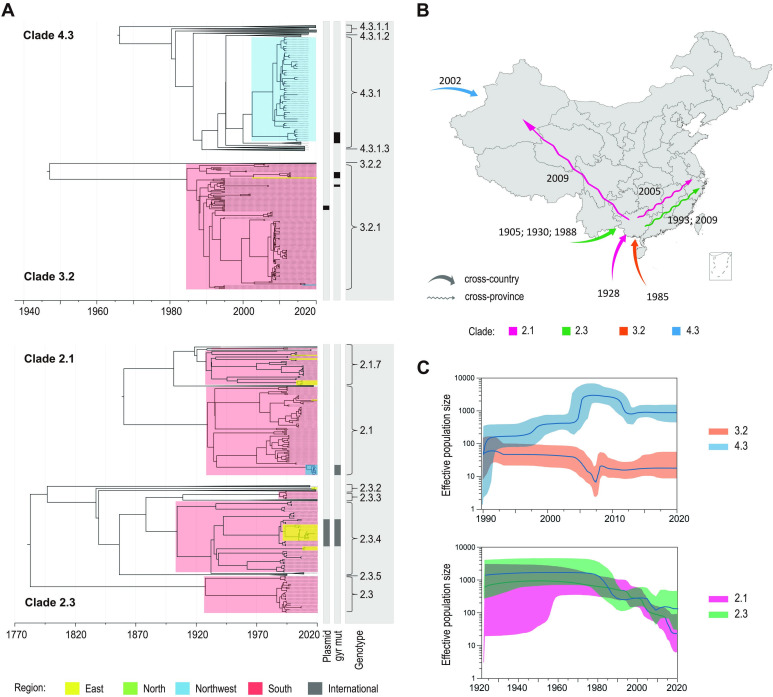
“Long-cycle” transmission of four dominant clades in China. (**A**) Maximum clade credibility tree (reconstructed using BEAST2) with the antimicrobial-associated metadata. The Chinese *S*. Typhi isolates from four regions are highlighted by boxes in different colors. The branches comprising international isolates are collapsed into triangles in gray. The right columns indicate the presence of plasmid and *gyr* mutations as well as the detailed genotypes. (**B**) Transmission routes and time inferred by BEAST analysis. Two kinds of arrows show transmission events from abroad and between provinces. The numbers near the arrows indicate the estimated year of the putative transmission events. (**C**) Bayesian skyline plots show the historical changes in the effective population size of the four clades. Lines and shadings indicate the medians and the 95% HPD intervals of estimated effective population sizes. The *x*-axis shows the time, and the *y*-axis indicates the population size.

The clades 2.1, 2.3, 3.2, and 4.3 outnumbered the others. They were frequently recovered from localized provinces over 4 years, indicating their long-term establishment as local reservoirs. To investigate the dates of emergence and geographical transfer of these clades, we verified their temporal structure (Fig. S6) and inferred their timed phylogenies for four major clades (Fig. 3).

The profile of geographical origin divided the four clades into two categories (single origin or multiple origin) (Fig. 3A; Fig. S7). The Chinese 3.2 and 4.3 isolates both formed a single branch that separated from the international isolates, indicating a single introduction into China. In contrast, the Chinese 2.1 and 2.3 isolates formed several branches that mixed with international isolates, suggesting that the two clades were introduced into different provinces of China in multiple waves. Notably, 2.3 was found to be repeatedly transmitted into the Guangxi province (in southern China) (Fig. 3A and B). This indicated that, Guangxi, a frontier of Chinese civilization throughout its history and a tourist destination in recent decades, served as a transmission hotspot: the internationally common clones were initially imported into Guangxi and later spread to eastern and northern China (Fig. 3B). Coincidentally, 2.1 and 2.3 entered China much earlier than 3.2 and 4.3. In particular, the establishment of 4.3 as an endemic clone in northwestern China could be traced back to 2002, coinciding with the time that local Muslims frequented the Hajj pilgrimage in the Middle East.

Bayesian estimation of the bacterial population showed a remarkable decline of 2.1, 2.3 and 3.2 since the 1990s (Fig. 3C). Only 4.3 underwent a growth of population, which corresponded to the global dissemination of this lineage.

### Recent “short-cycle” transmission within Chinese provinces

**Fig 4:**
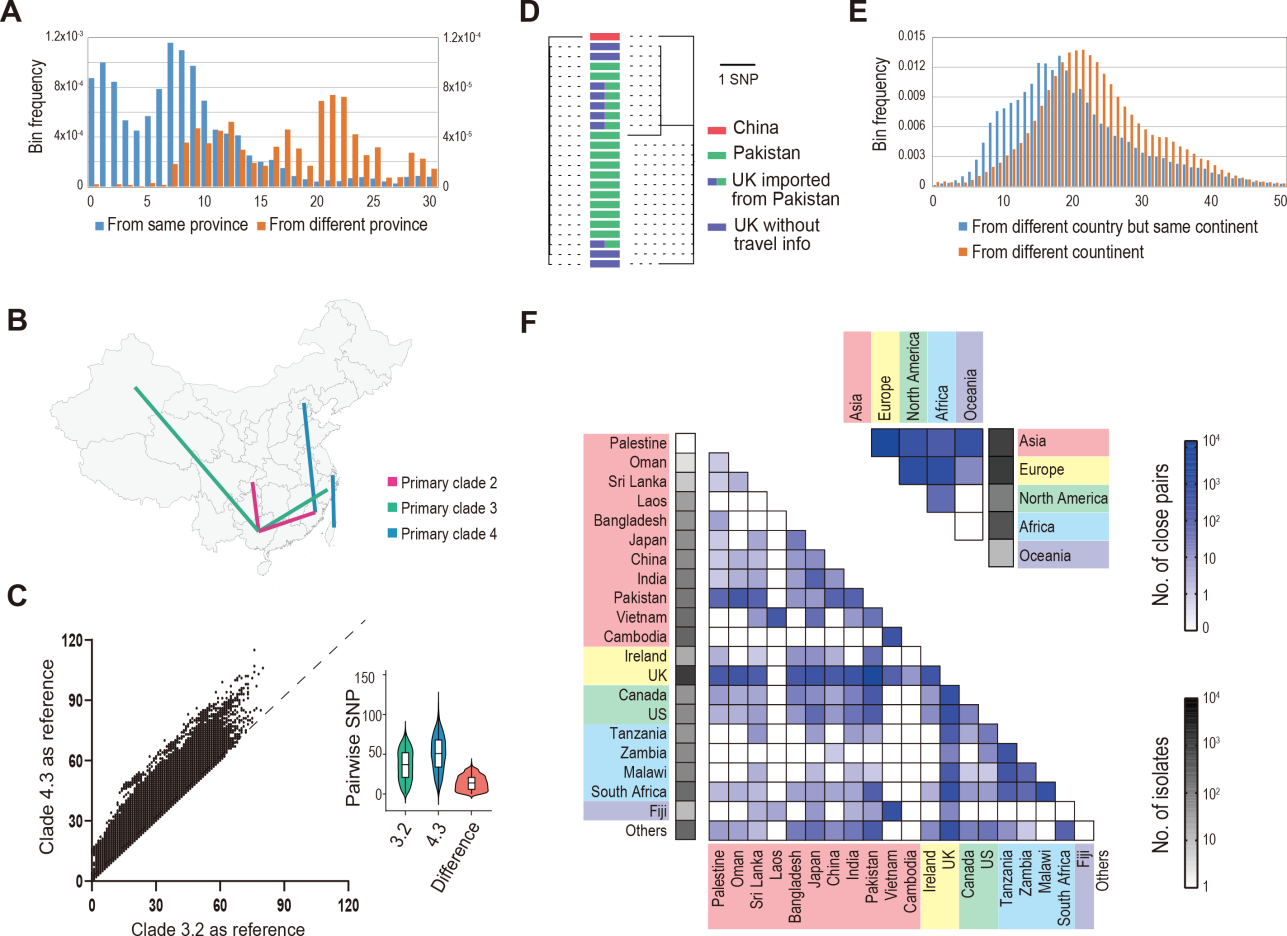
“Short-cycle” (or travel-associated) transmission of *S*. Typhi. (**A**) Histogram of cgSNP distance for the Chinese *S*. Typhi isolates collected from the same province (left *y*-axis) and different provinces (right *y*-axis). The *x*-axis indicates the number of cgSNPs. (**B**) Putative transmission events across provinces in China. Each line indicates a pair of *S*. Typhi isolates with pairwise distance <5 cgSNPs, indicating a putative transmission event. (**C**) Comparison of cgSNP distance between 4.3 isolates derived from different reference genomes as illustrated by a scatter plot and a violin plot, respectively. In the scatter plot, each dot indicates a pair of isolates, with its *x*-axis indicating the cgSNP distance calculated using the genome of strain CT18 (belonging to 3.2) as the reference and its *y*-axis using strain ERL052042 (accession number GCF_001088345, belonging to 4.3) as the reference. In the violin plot, the blue and green violins illustrate the distribution of the two sets of distance; and the red indicates their difference. (**D**) Example from the Chinese 4.3 isolate 458 (collected from Shanghai city) as well as the isolates of its closest genome match. Using CT18 strain as the reference, the left ML tree shows isolate 458 was one cgSNP distant from the international isolates (from the UK and Pakistan). Using ERL052042 as the reference, the right ML tree shows isolate 458 was one to three cgSNPs distant from these international isolates, but most of the international isolates still have the identical cgSNP profile, i.e., they cannot be distinguished from each other by their pairwise cgSNPs. (**E**) Histogram of cgSNP distance for 4.3 isolates collected from different countries and continents. The *x*-axis indicates the number of cgSNPs. (**F**) Genomic relatedness of 4.3 isolates worldwide as revealed by their pairwise cgSNP distance. The heatmaps show the numbers of close cgSNP matches (i.e., <5 cgSNPs) between countries and between continents. Countries with the same shade color belong to the same continents.

As travel-associated cases of *S*. Typhi infections have attracted increasing attention in developed countries (24), we examined putative travel-associated events in China based purely on genome similarity analysis. Except for 4.3, practically all *S*. Typhi isolates in China had their genetically closest relatives collected from the same province. This finding supports the prevalence of regional endemics in China. Notably, all Chinese 4.3 isolates are 4.3.1. We compared the histogram of the pairwise genetic distance of the isolates within the same province and between different provinces. The former showed a trough between two peaks at five cgSNPs, whereas the latter rose starting from five cgSNPs (Fig. 4A). Based on these findings, we used five cgSNPs as the threshold to determine if the compared isolates belonged to the same episode, resulting in the identification of six instances of cross-province transmission (Fig. 4B). Among them, four involved Guangxi, again demonstrating that Guangxi province has the most frequent within-China transmission events.

The 4.3 isolates collected from eastern China were not genetically closer to those from northwestern China but rather to international isolates (Fig. S7), providing strong evidence of their travel-associated nature. Nevertheless, as the Chinese 4.3 isolates showed an equally small distance from the international isolates from different countries, determining the specific country of origin was challenging. For example, an isolate from Shanghai was diverged from many of the UK and Pakistan isolates by a single cgSNP (Fig. 4D). We attributed this tiny genetic distance to a substantially lower within-clade nucleotide diversity of 4.3 than the other clades (Fig. S8). The calculation of cgSNPs employed the strain CT18 (belonging to 3.2) as the reference, which led to a loss of informative sites within the genomic segments specific to 4.3. Then, we switched to a 4.3 genome as the reference to increase genomic tracing resolution. This alternation expanded the core genome (i.e., the cgSNP searching range) from 3.97 to 4.51 Mb, and the average number of pairwise cgSNPs among 4.3 isolates increased from 36 to 51, indicating a 41.7% increase in resolution (Fig. 4C). Despite the improvement, several of the international isolates in the preceding example still exhibited the identical cgSNP profile (Fig. 4D). Out of the nine UK isolates that resembled the Shanghai isolate, six were imported from Pakistan via international travel (25, 26), explaining their genomic similarity. However, the remaining three lacked information on whether they were imported and from which countries they originated, therefore, their origin in Pakistan cannot be fully confirmed.

Expanded examination to global 4.3 showed the pattern of <5 cgSNP distance for the isolates from different countries of the same continent was, in general, similar to that from different continents, suggesting intercontinental transmission was almost as common as international transmission (Fig. 4E). The most frequent international transmission appears to occur between the UK and Pakistan; the most frequent intercontinental transmission between Europe and Asia (Fig. 4F).

### Phenotypic advantages of 4.3.1 over other lineages

**Fig 5:**
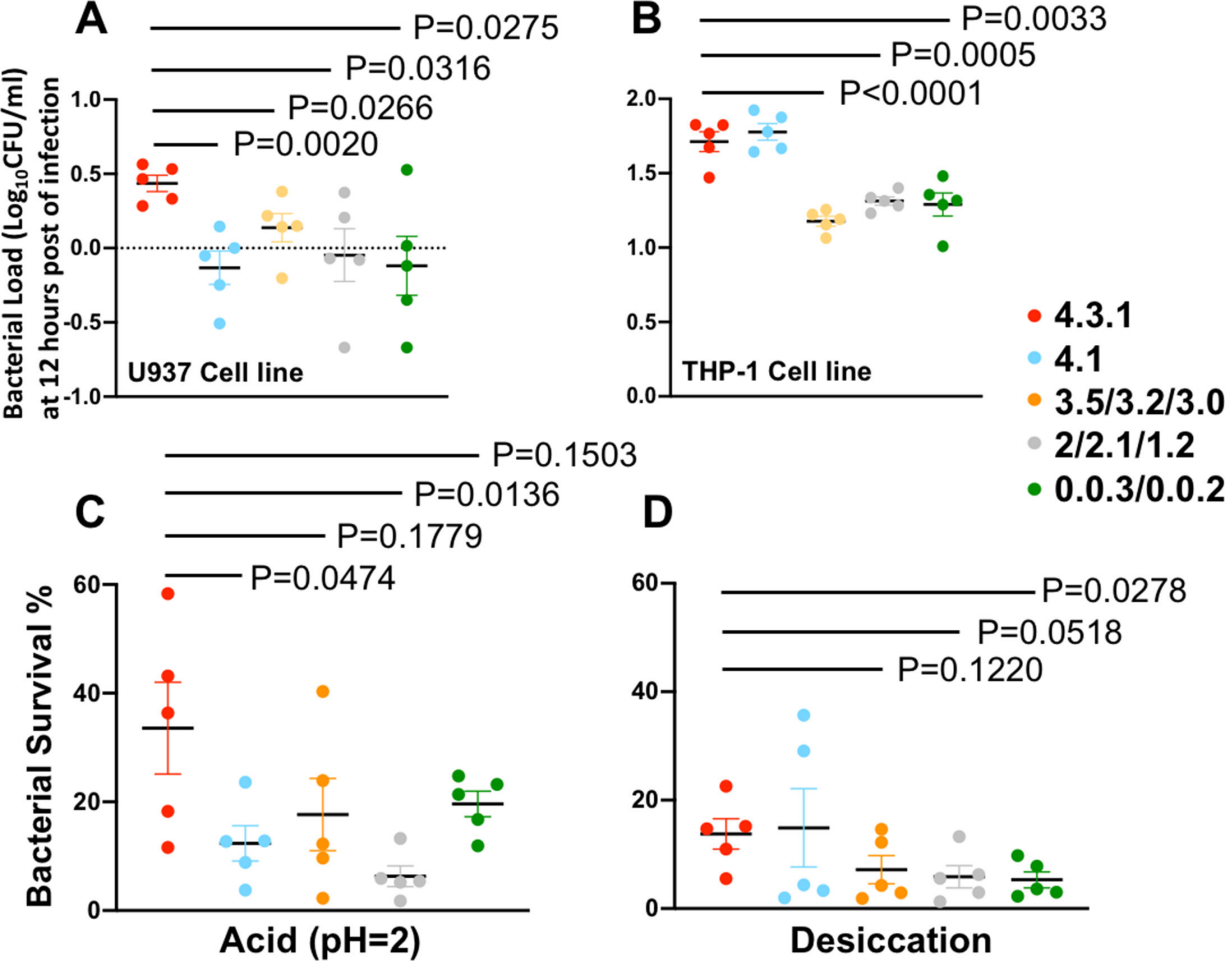
*In vitro* phenotypic comparison between 4.3.1 and other clades. Cellular invasion assays in the U937 cell line (**A**) and THP-1 cell line (**B**). The *y*-axis scale represents the log_10_ value of the ratio of detected bacteria at 12 h when compared with the initial bacteria that successfully invaded. *In vitro* tolerance assays with acid (pH = 2) (**C**) and desiccation (**D**). The *y*-axis scale represents the percentage of detected bacteria compared to the initial inoculation. Four groups of clinical isolates (Table S4) were chosen based on their lineage origin and availability in the current collection. The unpaired two-tailed *t* test was performed to assess the difference between groups with the calculated *P* value.

To understand the advantage of 4.3.1 as a pandemic clone, we selected strains to represent *S*. Typhi clades of different genetic backgrounds from our Chinese collections and conducted a series of *in vitro* assays (Table S4). In general, the 4.3.1 isolates exhibited significantly better intercellular survival abilities than isolates from the primary clades 0, 2, and 3, within both the human myeloid leukemia cell line (U937) (Fig. 5A) and human monocytic leukemia cell line (THP-1) (Fig. 5B) at 12-h post-infection. Compared with a close relative 4.1, 4.3.1 also shows a better survival rate within U937 cells. Regarding tolerance to acid (Fig. 5C) and desiccation (Fig. 5D), we also observed that 4.3.1 isolates have better survival rates, even though the *P* value did not always reach the significant threshold. However, there are no dramatic differences in biofilm formation among lineages under the four examined conditions (Fig. S9). Interestingly, all the isolates show strong biofilm formation capabilities under swine bile salt medium with anaerobic conditions.

## DISCUSSION

Here, we conducted, for the first time, a genomic epidemiology study of *S*. Typhi infection in China and found that both the “long-cycle” and “short-cycle” transmission modes commonly co-exist, providing striking regional heterogeneity. Most infections in eastern China resulted from “short-cycle” transmission, as revealed by their close match with international isolate genomes. This finding can be explained by the close connection of the populace in this region with other countries, such as southern Asia. By contrast, isolates from the other provinces displayed explicit geographical restrictions, suggesting that infections are attributable to the long-term circulation of the locally endemic clones. Dated phylogeny analysis further uncovered that endemic Chinese clones were not entirely indigenous but were, and continue to be, imported for several hundreds of years. For example, *S*. Typhi in northwestern China may well have been imported by frequent pilgrims from the Middle East, whereas the *S*. Typhi found in southern China almost certainly originated in adjacent Southeast Asia, a known reservoir for global typhoid fever (4). An interesting question is why *S*. Typhi imported to southern and northwestern China eventually developed into endemic clones, whereas those imported to eastern China only caused sporadic cases. Likewise, 4.3 imported by travelers to European countries has not created endemic disease, whereas importation to Africa via the same route, i.e., traveling public, has established a local dominant clone (27).

It has been recognized that climate and hydrological systems both play pivotal roles in the transmission of typhoid fever (2, 28 – 30). Northwestern and southern China are warm and damp, suitable for *Salmonella* growth. Furthermore, in the rural areas of these regions, local people utilize river or well water for cooking and cleaning (31, 32), similar to the endemic typhoid regions of South Asia, Southeast Asia, and Africa. As well as the popularity of foreign travel in eastern China, further multi-faceted contributions led to dual transmission modes in China. These may explain the relatively low correlation of typhoidal incidence with the GDP and tourist numbers (Fig. 1C).

In the era of the global village, genome-based epidemiology has been increasingly employed to reconstruct transmission links for many infectious diseases, which is built on the assumption that the natural origin of the disease tends to come from the region with the closest genome match (33, 34). This assumption has a premise that bacterial genomes must show robust geographical clustering, with considerable distance between isolates from different regions. This premise holds for most bacterial pathogens, including *S*. Typhi 2.1, 2.3, and 3.2 in China. By comparison, predicting the geographic origin of 4.3 genomes is much more challenging. The within-clade cgSNP distance is small, and frequent international transfer has distorted geographical clustering. Last but not least, the biased data sources of public databases have impeded global tracking investigations. Many of the developing countries, which are the real sources of typhoid disease, have submitted very few genomic data sets, probably due to the cost of establishing sequencing-based surveillance systems. Whereas the UK holds the largest data set of sequenced isolates, making it more likely to be the country with the closest genome match to any query isolates. Consequently, when the travel history of the UK patients is missing, the UK is easy to be misidentified as the country of origin.

As proposed here, three measures may help address the above issues. First, a hierarchical typing scheme, e.g., using a specific reference genome for 4.3.1 isolates, has been shown to increase the resolution. Second, tracing the exact source country should depend not only on the count of cgSNP distance but also on the topology of the dated phylogeny or even more complicated spatial-temporal modeling that comprises all the closely related isolates (24). Third, well-integrated databases are urgently required, which include not only the site of isolation/sequencing but also the site of infection (or rather the travel history). Last, increasing the density of isolate collection and genome sequencing from developing countries, including China, would effectively avoid sampling bias and help depict an accurate transmission route.

Over the last three decades, intercontinental transmission and circulation have been dominated by 4.3.1 or called H58. In line with this study, we found that the short-cycle international transmission in China was exclusively attributed to 4.3.1. MDR was once thought to drive the worldwide expansion of 4.3.1 (12, 35), but MDR is also present in other clades. Our bile resistance assay refuted the hypothesis that 4.3.1 has higher bile resistance benefiting persistence in the gallbladder in asymptomatic patients, which is in line with a previous report that *S*. Typhi carriage is not restricted to any particular genotype (36). Interestingly, we found an improved ability of 4.3.1 to resist a low pH environment, which might allow easier access to the intestine. We also found that 4.3.1 can better survive and replicate in macrophages. While this ability assists 4.3.1 to elude the host immune response, it might also benefit 4.3.1 living within amoebae in the environment. Similarly, the improved capability of desiccation tolerance may likely contribute to the environmental persistence of 4.3.1. Notably, 4.3.1 may acquire these capabilities in a stepwise manner. Our results showed that 4.3.1 has comparable desiccation tolerance and intracellular survival abilities in THP-1 compared to its close relative 4.1. But 4.3.1 performs better in acid tolerance and survival in U937, suggesting 4.3.1 has evolved the latter two capabilities after it diverged from 4.1. Nevertheless, all the examined results are based on Chinese clinical isolates, further investigations with an extensive diversified collection in the global community are highly warranted.

In conclusion, we provided the largest genomic data set from China for global surveillance and AMR assessment for one of the oldest killer diseases in the world. More importantly, our nationwide study revealed a novel dual-transmission mode with distinct regional features, which also correlated with the climate, and social-economic parameters of those regions in China. A notable historic “long-cycle,” mainly endemic in inter-mainland China, is associated with the provinces that require improvements to hygiene, particularly water sanitation. Recent “short-cycle” transmission, mainly in more advanced socioeconomic areas in eastern China, is related to intensified global travel and linked with the MDR pandemic 4.3.1. Furthermore, we provided new biological evidence of tolerance capabilities that may favor typhoid transmission. The gained knowledge in this study would tailor the policy with precise interventions according to distinct transmission hotspots and modes in different regions.

## MATERIALS AND METHODS

### Data on *S*. Typhi incidence and correlation analysis

The annual incidence of *S*. Typhi infection was obtained from the Data Center of China Public Health Science (https://www.phsciencedata.cn/). The temperature data were retrieved from the website of the National Oceanic and Atmospheric Administration (www.noaa.gov). The precipitation data were recovered from the software WheatA (http://www.wheata.cn/). The latitude data were obtained from the Baidu Baike database (https://baike.baidu.com/), using the provincial capital city to represent the province. The GDP data were retrieved from the OuWei database (https://www.ovo.com.cn/). The tourism data were obtained from the Baidu Wenku database (https://baike.baidu.com/). All the collected data sets are in Table S1. The Spearman correlation between the *S*. Typhi incidence and the variables was calculated with R software v4.0.3.

### Bacterial isolates and WGS

A total of 705 *S*. Typhi isolates were recovered between 1960 and 2020, originating from 17 provinces in China (Table S2). The isolates yielded from humans (*n* = 690) or water (*n* = 10). Human samples included blood (*n* = 616), feces (*n* = 36), marrow (*n* = 4), anal swab (*n* = 1), bile (*n* = 1), body fluid (*n* = 1), urine (*n* = 1), and unknown type (*n* = 30). Water samples included effluent (*n* = 6), pipe water (*n* = 3), and well water (*n* = 1). The sources for the remaining samples included environment (toilet, *n* = 2) and unknown source (*n* = 3).

Most of the isolates were collected by several major CDCs around the country, including Zhejiang CDC, Shanghai CDC, and Shandong CDC. The First Affiliated Hospital of Zhejiang University School of Medicine contributed to assembling the Bacterial Resistant Investigation Collaborative System collection, which collected bloodstream patients from 23 tertiary hospitals and 29 non-tertiary hospitals covering 18 provinces of mainland China. These organizations collected the isolates from subordinate local CDCs under an active routine surveillance program. Human samples were collected from patients with clinical diagnosis (*n* = 653), confirmed carrier cases (*n* = 1), and people with unknown status of infection (*n* = 36).

Total genomic DNA was extracted from the *S*. Typhi using the TIANamp genomic DNA extraction Kit (RT405, TIANGEN BIOTECH, China) and subjected to WGS using the Illumina HiSeq 2000 or HiSeq 2500 platforms (Illumina, San Diego, CA, USA) to generate 150 bp paired-end reads. *De novo* assembly of genome sequences was performed using SPAdes v3.12.0 (37). Serotyping was further confirmed by SeqSero2 v1.1.1 and SISTR v1.0.2 (38, 39).

### Genotyping and phylogenetic analyses

To provide global context, a total of 5,190 publicly available *S*. Typhi genomes retrieved from the Enterobase *Salmonella* database and NCBI genome database (until 9 May 2021, including 26 genomes of Chinese isolates and 5,164 international isolates with the genomes) were included for analysis in addition to the genomes sequenced in this study (Tables S2 and S3). The cgSNPs were classified by mapping the assemblies of all query genomes to the reference sequence of *S*. Typhi strain CT18 (accession no: AL513382) with SNIPPY software v4.4.4 (40). The recombinant regions were removed using Gubbins v2.4.1, inset in SNIPPY (41). From the identified SNPs, a subset of 68 was assigned *S*. Typhi isolates to previously defined lineages according to the existing extended *S*. Typhi genotyping framework with GenoTyphi v1.9.1 (9). The genomes belonging to 4.3 were also mapped to the reference sequence of 4.3 strain ERL052042 (accession no: GCF_001088345) to obtain a second set of cgSNP using the same analytical procedures as the above and below methods.

Concatenated cgSNPs were aligned to build maximum likelihood (ML) phylogenetic trees using IQ-Tree v2.2.0 with default parameters (42). The ML trees were displayed and annotated using iTOL v6 (43). The population structure of the global *S*. Typhi collection was also analyzed by hierarchical Bayesian Analysis of Population Structure v6.0 (44), a phylogeny-free population genetics approach; six levels of clustering were performed in the hierarchy, and a prior upper boundary of 80 clusters was set.

Pairwise genetic distances (the difference in the number of cgSNPs) within and between SCs were calculated from the cgSNP alignment using the dist.dna function in the R package ape v5.4.1.

### Temporal and phylogeographical analysis

To verify the temporal structure of the dominant four clades in China, we first used TempEst v1.5.1 to assess temporal structure by conducting a regression analysis of the root-to-tip branch distances of the ML tree as a function of sampling date (45). A date randomization test was also conducted by the TipDatingBeast v1.1 R package (46), in which 20 times of analysis were repeated (47). Then, the four clades were subjected to model-based most recent common ancestor dating and phylogeographical reconstruction using Bayesian evolutionary analysis by sampling trees (BEAST) v2.6.7 (48). To understand international transmission events, the representatives of international isolates were also included in this analysis. We identified clusters of closely related isolates with the same sample source, isolation year, and country of isolation and differed by less than five cgSNPs. One representative isolate was randomly selected from each cluster and was kept in the analysis.

The BEAST analysis was performed on alignments of concatenated cgSNPs following previously described procedures (11). Briefly, the GTR+Γ4 substitution model, Bayesian skyline model, and relaxed log-normal molecular clock were chosen; the chain length was 100 million, with sampling at every 10,000 iterations. Maximum clade credibility trees were generated using TreeAnnotator v2.6.7. Figtree v1.4.3 was used to annotate and visualize the tree. The effective population size was estimated by using Tracer v1.7.1.

### Identification of AMR-associated determinants

ABRicate v0.8 and the CGE ResFinder database were used to investigate AMR-associated genes (49). Plasmid replicons were identified using ABRicate v0.8 and PlasmidFinder database (50). Point mutations in the quinolone-resistance determining region of genes *gyrA*, *gyrB*, and *parC*; and the *acrB*- mutations associated with azithromycin resistance were detected using ResFinder v4.1.0 and PointFinder database (49).

### Rational choice of Chinese bacterial strains for *in vivo* assays

The strains used in this study are listed in Table S4. These strains were chosen to better reflect significant Chinese clades, with the purpose of comparative analysis for pandemic clone 4.3.1. Among three major primary clades (2, 3, and 4), two to three additional major genotypes of each primary clade were considered, including 2.1, 2, and 2.3.4 from primary clade 2, 3.2.1, 3.5, and 3.0.1 from primary clade 3, and 4.3.1 and 4.1 from primary clade 4. For other minor primary clades, two genotypes with clearly known classification names (0.0.3 and 0.0.2) were selected for primary clade 0, and we used the only available strain representing primary clade 1. For lineage 4.3.1, each of its three genotypes (4.3.1, 4.3.1.2, and 4.3.1.1P1) was highly linked to a geographical region of China: subtype 4.3.1 was mainly from Northwestern China (51/53, 96.2%), lineage 4.3.1.1 was mainly from Eastern China (6/6, 100%), and lineage 4.3.1.2 was mainly from Eastern China (5/5, 100%). Additionally, diversities of years of bacterial isolation and ages/sexes of corresponding patients were both fully considered for a better-represented isolate. Notably, our choice of strains is highly limited to our Chinese collection. The above strains were recovered and routinely grown in Luria Broth (LB) medium.

### Acid stress assay

Overnight cultures of 26 strains were transferred to fresh LB medium at a ratio of 1:100 and cultured at 37°C, 220 r/min to OD_600nm_ 0.4–0.5 (10^9^ CFU/mL). Plate-count (PC) method was used to record the initial bacterial concentration. Appropriate diluent (10 µL) of the original inoculum was streaked on an LB plate, and incubated at 37°C for 14–18 h (three replicates were performed for each sample). NaCl (Sinopharm Chemical Reagent Co., Ltd), NaOH, and HCl (AR, SCR/HuShi) were used to prepare physiological saline (pH = 2). A 10 µL resuspension solution mentioned above was 10× gradient diluted in physiological saline (pH = 2) to 10^7^ CFU/mL and then placed at 37°C for 10 min. The plate counting method was used for recording the number of viable bacteria. The survival rate of the bacteria was calculated (the number of viable numbers/the number of initial bacteria × 100%). Data were analyzed using GraphPad Prism v9.0 software. The unpaired two-tailed *t* test was performed to assess the difference between groups.

### Desiccation suppression assay

Overnight culture (16 h at 37°C 220 r/min) of 26 strains was prepared and adjusted to OD_600nm_ value 1.0 (10^9^ CFU/mL). A total of 50 µL inoculum was added to a 96-well plate. And the plate was placed in a sealed box containing saturated potassium acetate (Sinopharm Chemical Reagent Co., Ltd) at 37°C for 24 h. After that, the plate counting method was performed (three replicates for each strain) to calculate the survival rate of the bacteria. The unpaired two-tailed *t* test was performed to assess the difference between groups.

### Biofilm assay

Biofilm assay was performed in a similar way to our previously reported (51, 52). As mentioned in the desiccation suppression assay, overnight culturing was carried out in LB broth. Bacteria were transferred at a ratio of 1:100 into LB medium supplemented with Swine Bile Salt (0.5%), Ox Bile Salt (0.1%), and No.3 Bile Salt (0.1%), respectively. These bile salts were purchased in Shanghai Bo Microbiology Technology Co., Ltd. After thoroughly mixing, 200 µL inoculum was seeded in each 96-well plate in quintuplicate. *Escherichia coli* strain ATCC25922 and *P. aeruginosa* ATCC27853 were used as the positive control. The inoculated plate was incubated at 28°C for 24 h under aerobic and anaerobic conditions, respectively. After 24 h, the wells of the inoculated plate were washed gently with ddH_2_O for three times. After drying, each well was stained with 200 µL of crystal violet (Sinopharm Chemical Reagent Co., Ltd) at room temperature for 20 min. After staining, each well was washed gently with ddH_2_O for three times and 200 µL f 75% ethanol (Sinopharm Chemical Reagent Co., Ltd) was added. The dye was wholly dissolved and decolorized by shaking for 20 min. Before measuring results, the product of decolorization was transferred to a new microtiter plate. OD_600nm_ value of each stained well was measured using a microtiter-plate reader of the Tecan spectrophotometer. The unpaired two-tailed *t* test was performed to assess the difference between groups.

### Intracellular survival assay

Cellular assays referred to methods previously reported (53, 54). RPMI1640 (Gibco) containing 5% fetal bovine serum (FBS; purchased from Biowest) was applied as a cell culture medium for recovering and culturing. Cells were grown in an incubator maintained at 37°C with 5% CO_2_ and passaged to the fifth generation. The THP-1 cells were diluted to 5  ×  10^4^ cells/mL by RPMI1640 supplemented with 5% FBS and 50  ng/mL PMA for 48 h. U937 cells were diluted to 5  ×  10^4^ cells/mL by RPMI1640 supplemented with 5% FBS and 25 ng/mL PMA for 48 h to prepare the cell lines. The culture medium was discarded and replaced with RPMI1640 (containing 5% FBS) for an additional 12 h culture.

The intracellular survival assay was performed as follows: (i) the overnight culturing was carried out in LB broth as mentioned in the desiccation suppression assay; (ii) the inoculum was diluted with RPMI1640, and a volume of 200 µL was transferred to the well for infecting cells with an MOI of 50:1 for 1 h at 37°C, while plate counting method was performed for recording concentration of initial bacteria in the inoculum; (iii) the cells were washed three times with warm RPMI1640 to remove extracellular bacteria; (4) gentamicin was added to the RPMI1640 medium (containing 5% FBS) at a final concentration of 100 µg/mL and the culture was incubated for 1 h to kill extracellular bacteria; (5) RPMI1640 medium (containing 5% FBS) supplemented with 10 g/mL gentamicin (Sangon Biotech) was used for the new culture after discarding the old medium, and the new culture was incubated at 37°C with 5% CO_2_ for 0 and 12 h, respectively, while the cell lysis were subjected to plate couting at each time point; and (6) the survival rate of the bacteria was calculated (the number of viable bacteria at 12 h/the number of initial bacteria that successfully invaded at 0 h × 100%). The unpaired two-tailed t test was performed to assess the difference between groups.

## Data Availability

Raw sequence data/genome assemblies are available in the NCBI SRA database under accession PRJNA875300, the *Salmonella* public database of Enterobase (55), or the NCBI Genome database. Tables S2 and S3 list the accession number for each isolate used in this study.

## SUPPLEMENTAL MATERIAL

Fig. S2 mbio.01333-23-s0001.tif Geographic analysis.

Fig. S3 mbio.01333-23-s0002.tif Clonal replacement.

Fig. S4 mbio.01333-23-s0003.tif Age and sex analysis.

Fig. S5 mbio.01333-23-s0004.tif Plasmid analysis.

Fig. S6 mbio.01333-23-s0005.pdf Assessment of the temporal structure of clades.

Fig. S7 mbio.01333-23-s0006.pdf ML tree.

Fig. S8 mbio.01333-23-s0007.tif Pairwise SNP.

Fig. S9 mbio.01333-23-s0008.tif Biofilm analysis.

Supplemental material mbio.01333-23-s0009.pdf Supplemental legends and Fig. S1.

Supplemental tables mbio.01333-23-s0010.xlsx Tables S1 to S4.

ASM does not own the copyrights to Supplemental Material that may be linked to, or accessed through, an article. The authors have granted ASM a non-exclusive, world-wide license to publish the Supplemental Material files. Please contact the corresponding author directly for reuse.

## References

[1] Stanaway JD , Reiner RC , Blacker BF , Goldberg EM , Khalil IA , Troeger CE , Andrews JR , Bhutta ZA , Crump JA , Im J , Marks F , Mintz E , Park SE , Zaidi AKM , Abebe Z , Abejie AN , Adedeji IA , Ali BA , Amare AT , Atalay HT , Avokpaho E , Bacha U , Barac A , Bedi N , Berhane A , Browne AJ , Chirinos JL , Chitheer A , Dolecek C , El Sayed Zaki M , Eshrati B , Foreman KJ , Gemechu A , Gupta R , Hailu GB , Henok A , Hibstu DT , Hoang CL , Ilesanmi OS , Iyer VJ , Kahsay A , Kasaeian A , Kassa TD , Khan EA , Khang Y-H , Magdy Abd El Razek H , Melku M , Mengistu DT , Mohammad KA , Mohammed S , Mokdad AH , Nachega JB , Naheed A , Nguyen CT , Nguyen HLT , Nguyen LH , Nguyen NB , Nguyen TH , Nirayo YL , Pangestu T , Patton GC , Qorbani M , Rai RK , Rana SM , Ranabhat CL , Roba KT , Roberts NLS , Rubino S , Safiri S , Sartorius B , Sawhney M , Shiferaw MS , Smith DL , Sykes BL , Tran BX , Tran TT , Ukwaja KN , Vu GT , Vu LG , Weldegebreal F , Yenit MK , Murray CJL , Hay SI . 2019. The global burden of typhoid and paratyphoid fevers: a systematic analysis for the Global Burden of Disease Study 2017. Lancet Infect Dis 19:369–381. doi:10.1016/S1473-3099(18)30685-6 30792131 PMC6437314

[2] Gauld JS , Olgemoeller F , Heinz E , Nkhata R , Bilima S , Wailan AM , Kennedy N , Mallewa J , Gordon MA , Read JM , Heyderman RS , Thomson NR , Diggle PJ , Feasey NA . 2022. Spatial and genomic data to characterize endemic typhoid transmission. Clin Infect Dis 74:1993–2000. doi:10.1093/cid/ciab745 34463736 PMC9187325

[3] Meiring JE , Shakya M , Khanam F , Voysey M , Phillips MT , Tonks S , Thindwa D , Darton TC , Dongol S , Karkey A , Zaman K , Baker S , Dolecek C , Dunstan SJ , Dougan G , Holt KE , Heyderman RS , Qadri F , Pitzer VE , Basnyat B , Gordon MA , Clemens J , Pollard AJ , STRATAA Study Group . 2021. Burden of enteric fever at three urban sites in Africa and Asia: a multicentre population-based study. Lancet Glob Health 9:e1688–e1696. doi:10.1016/S2214-109X(21)00370-3 34798028 PMC8609278

[4] Carey ME , MacWright WR , Im J , Meiring JE , Gibani MM , Park SE , Longley A , Jeon HJ , Hemlock C , Yu AT , Soura A , Aiemjoy K , Owusu-Dabo E , Terferi M , Islam S , Lunguya O , Jacobs J , Gordon M , Dolecek C , Baker S , Pitzer VE , Yousafzai MT , Tonks S , Clemens JD , Date K , Qadri F , Heyderman RS , Saha SK , Basnyat B , Okeke IN , Qamar FN , Voysey M , Luby S , Kang G , Andrews J , Pollard AJ , John J , Garrett D , Marks F . 2020. The surveillance for enteric fever in Asia Project (SEAP), Severe Typhoid fever surveillance in Africa (SETA), surveillance of enteric fever in India (SEFI), and strategic typhoid alliance across Africa and Asia (STRATAA) population-based enteric fever studies: a review of methodological similarities and differences. Clin Infect Dis 71:S102–S110. doi:10.1093/cid/ciaa367 32725221 PMC7388711

[5] Im J , Islam MT , Ahmmed F , Kim DR , Islam Khan A , Zaman K , Ali M , Marks F , Qadri F , Kim J , Clemens JD . 2021. Can existing improvements of water, sanitation, and hygiene (WASH) in urban slums reduce the burden of typhoid fever in these settings? Clin Infect Dis 72:e720–e726. doi:10.1093/cid/ciaa1429 32964216

[6] Holt KE , Parkhill J , Mazzoni CJ , Roumagnac P , Weill F-X , Goodhead I , Rance R , Baker S , Maskell DJ , Wain J , Dolecek C , Achtman M , Dougan G . 2008. High-throughput sequencing provides insights into genome variation and evolution in Salmonella Typhi. Nat Genet 40:987–993. doi:10.1038/ng.195 18660809 PMC2652037

[7] Kidgell C , Reichard U , Wain J , Linz B , Torpdahl M , Dougan G , Achtman M . 2002. Salmonella Typhi, the causative agent of typhoid fever, is approximately 50,000 years old. Infect Genet Evol 2:39–45. doi:10.1016/s1567-1348(02)00089-8 12797999

[8] Pan H , Jia C , Paudyal N , Li F , Mao J , Liu X , Dong C , Zhou K , Liao X , Gong J , Fang W , Li X , Kehrenberg C , Yue M . 2022. Comprehensive assessment of subtyping methods for improved surveillance of foodborne Salmonella. Microbiol Spectr 10:e0247922. doi:10.1128/spectrum.02479-22 36194132 PMC9602514

[9] Wong VK , Baker S , Connor TR , Pickard D , Page AJ , Dave J , Murphy N , Holliman R , Sefton A , Millar M , Dyson ZA , Dougan G , Holt KE , International Typhoid Consortium . 2016. An extended genotyping framework for Salmonella enterica serovar typhi, the cause of human typhoid. Nat Commun 7:12827. doi:10.1038/ncomms12827 27703135 PMC5059462

[10] Dyson ZA , Holt KE . 2021. Five years of GenoTyphi: updates to the global Salmonella typhi genotyping framework. J Infect Dis 224:S775–S780. doi:10.1093/infdis/jiab414 34453548 PMC8687072

[11] Wong VK , Baker S , Pickard DJ , Parkhill J , Page AJ , Feasey NA , Kingsley RA , Thomson NR , Keane JA , Weill F-X , Edwards DJ , Hawkey J , Harris SR , Mather AE , Cain AK , Hadfield J , Hart PJ , Thieu NTV , Klemm EJ , Glinos DA , Breiman RF , Watson CH , Kariuki S , Gordon MA , Heyderman RS , Okoro C , Jacobs J , Lunguya O , Edmunds WJ , Msefula C , Chabalgoity JA , Kama M , Jenkins K , Dutta S , Marks F , Campos J , Thompson C , Obaro S , MacLennan CA , Dolecek C , Keddy KH , Smith AM , Parry CM , Karkey A , Mulholland EK , Campbell JI , Dongol S , Basnyat B , Dufour M , Bandaranayake D , Naseri TT , Singh SP , Hatta M , Newton P , Onsare RS , Isaia L , Dance D , Davong V , Thwaites G , Wijedoru L , Crump JA , De Pinna E , Nair S , Nilles EJ , Thanh DP , Turner P , Soeng S , Valcanis M , Powling J , Dimovski K , Hogg G , Farrar J , Holt KE , Dougan G . 2015. Phylogeographical analysis of the dominant multidrug-resistant H58 clade of Salmonella typhi identifies inter- and intracontinental transmission events. Nat Genet 47:632–639. doi:10.1038/ng.3281 25961941 PMC4921243

[12] Holt KE , Phan MD , Baker S , Duy PT , Nga TVT , Nair S , Turner AK , Walsh C , Fanning S , Farrell-Ward S , Dutta S , Kariuki S , Weill F-X , Parkhill J , Dougan G , Wain J . 2011. Emergence of a globally dominant IncHI1 plasmid type associated with multiple drug resistant typhoid. PLoS Negl Trop Dis 5:e1245. doi:10.1371/journal.pntd.0001245 21811646 PMC3139670

[13] Hendriksen RS , Leekitcharoenphon P , Lukjancenko O , Lukwesa-Musyani C , Tambatamba B , Mwaba J , Kalonda A , Nakazwe R , Kwenda G , Jensen JD , Svendsen CA , Dittmann KK , Kaas RS , Cavaco LM , Aarestrup FM , Hasman H , Mwansa JCL . 2015. Genomic signature of multidrug-resistant Salmonella enterica serovar typhi isolates related to a massive outbreak in Zambia between 2010 and 2012. J Clin Microbiol 53:262–272. doi:10.1128/JCM.02026-14 25392358 PMC4290967

[14] Argimón S , Nagaraj G , Shamanna V , Sravani D , Vasanth AK , Prasanna A , Poojary A , Bari AK , Underwood A , Kekre M , Baker S , Aanensen DM , Lingegowda RK . 2022. Circulation of third-generation cephalosporin resistant Salmonella Typhi in Mumbai, India. Clin Infect Dis 74:2234–2237. doi:10.1093/cid/ciab897 34626469 PMC9258936

[15] Carey ME , Jain R , Yousuf M , Maes M , Dyson ZA , Thu TNH , Nguyen Thi Nguyen T , Ho Ngoc Dan T , Nhu Pham Nguyen Q , Mahindroo J , Thanh Pham D , Sandha KS , Baker S , Taneja N . 2021. Spontaneous emergence of azithromycin resistance in independent lineages of Salmonella Typhi in northern India. Clin Infect Dis 72:e120–e127. doi:10.1093/cid/ciaa1773 33515460 PMC7935384

[16] Klemm EJ , Shakoor S , Page AJ , Qamar FN , Judge K , Saeed DK , Wong VK , Dallman TJ , Nair S , Baker S , Shaheen G , Qureshi S , Yousafzai MT , Saleem MK , Hasan Z , Dougan G , Hasan R . 2018. Emergence of an extensively drug-resistant Salmonella enterica serovar typhi clone harboring a promiscuous plasmid encoding resistance to fluoroquinolones and third-generation cephalosporins. mBio 9:e00105-18. doi:10.1128/mBio.00105-18 29463654 PMC5821095

[17] Song Q , Yang Y , Lin W , Yi B , Xu G . 2017. Epidemiological characteristics and clinical treatment outcome of typhoid fever in Ningbo, China, 2005-2014: pulsed-field GEL electorophoresis results revealing great proportion of common transmission sources. Jpn J Infect Dis 70:513–517. doi:10.7883/yoken.JJID.2016.434 28367881

[18] Wang J-F , Wang Y , Zhang J , Christakos G , Sun J-L , Liu X , Lu L , Fu X-Q , Shi Y-Q , Li X-M . 2013. Spatiotemporal transmission and determinants of typhoid and paratyphoid fever in Hongta District, Yunnan Province, China. PLoS Negl Trop Dis 7:e2112. doi:10.1371/journal.pntd.0002112 23516653 PMC3597484

[19] Yue M , Bai L , Song H , Fang W . 2021. Impacts of microbial food safety in China and beyond. Foodborne Pathog Dis 18:508–509. doi:10.1089/fpd.2021.29015.int 34403267

[20] Yan M , Li X , Liao Q , Li F , Zhang J , Kan B . 2016. The emergence and outbreak of multidrug-resistant typhoid fever in China. Emerg Microbes Infect 5:e62. doi:10.1038/emi.2016.62 27329848 PMC4932652

[21] Hu B , Hou P , Teng L , Miao S , Zhao L , Ji S , Li T , Kehrenberg C , Kang D , Yue M . 2022. Genomic investigation reveals a community typhoid outbreak caused by contaminated drinking water in China, 2016. Front Med (Lausanne) 9:753085. doi:10.3389/fmed.2022.753085 35308507 PMC8925297

[22] Wang Y , Lu D , Jin Y , Wang H , Lyu B , Zhang X , Huang Y , Shu G , Liu B , Lin C , Zhao H , Zhao M , Shen L , Gao Z , Zhang D , Wang Q , Qu M , Jia L . 2022. Extensively drug-resistant (XDR) Salmonella Typhi outbreak by waterborne infection - Beijing municipality, China, January-February 2022. China CDC Wkly 4:254–258. doi:10.46234/ccdcw2022.062 35433085 PMC9005487

[23] Carey ME , Dyson ZA , Ingle DJ , Amir A , Aworh MK , Chattaway MA , Chew KL , Crump JA , Feasey NA , Howden BP , Keddy KH , Maes M , Parry CM , Puyvelde SV , Webb HE , Afolayan AO , Anandan S , Andrews JR , Ashton PM , Basnyat B , Bavdekar A , Bogoch II , Clemens JD , da Silva KE , De A , de Ligt J , Diaz Guevara PL , Dolecek C , Dutta S , Watkins LF , Garrett DO , Godbole G , Gordon MA , Greenhill AR , Griffin C , Gupta M , Hendricksen R , Heyderman RS , Hooda Y , Hormazabal JC , Ikhimiukor OO , Iqbal J , Jacob JJ , Jenkins C , Jinka DR , John J , Kang G , Kanteh A , Kapil A , Karkey A , Kariuki S , Kingsley RA , Koshy RM , Lauer AC , Levine MM , Lingegowda RK , Luby SP , Mackenzie GA , Mashe TA , Msefula C , Mutreja A , Nagaraj G , Nagaraj S , Nair S , Naseri TK , Nimarota-Brown S , Njamkepo E , Okeke IN , Perumal SPB , Pollard AJ , Pragasam AK , Qadri F , Qamar FN , Rahman SIA , Rambocus SD , Rasko DA , Ray P , Robins-Browne R , Rongsen-Chandola T , Rutanga JP , Saha SK , Saha S , Saigal K , Sajib MSI , Seidman JC , Shakya J , Shamanna V , Shastri J , Shrestha R , Sia S , Sikorski MJ , Singh A , Smith AM , Tagg KA , Tamrakar D , Tanmoy AM , Thomas M , Thomas MS , Thomsen R , Thomson NR , Tupua S , Vaidya K , Valcanis M , Veeraraghavan B , Weill F-X , Wright J , Dougan G , Argimón S , Keane JA , Aanensen DM , Baker S , Holt KE , Global Typhoid Genomics Consortium Group Authorship . 2022. Global diversity and antimicrobial resistance of typhoid fever pathogens: insights from 13,000 Salmonella typhi genomes. medRxiv. doi:10.1101/2022.12.28.22283969 PMC10506625 37697804

[24] da Silva KE , Tanmoy AM , Pragasam AK , Iqbal J , Sajib MSI , Mutreja A , Veeraraghavan B , Tamrakar D , Qamar FN , Dougan G , Bogoch I , Seidman JC , Shakya J , Vaidya K , Carey ME , Shrestha R , Irfan S , Baker S , Luby SP , Cao Y , Dyson ZA , Garrett DO , John J , Kang G , Hooda Y , Saha SK , Saha S , Andrews JR . 2022. The international and intercontinental spread and expansion of antimicrobial-resistant Salmonella Typhi: a genomic epidemiology study. Lancet Microbe 3:e567–e577. doi:10.1016/S2666-5247(22)00093-3 35750070 PMC9329132

[25] Nair S , Chattaway M , Langridge GC , Gentle A , Day M , Ainsworth EV , Mohamed I , Smith R , Jenkins C , Dallman TJ , Godbole G . 2021. ESBL-producing strains isolated from imported cases of enteric fever in England and Wales reveal multiple chromosomal integrations of bla_CTX-M_-15 in XDR Salmonella Typhi. J Antimicrob Chemother 76:1459–1466. doi:10.1093/jac/dkab049 33704480

[26] Chattaway MA , Gentle A , Nair S , Tingley L , Day M , Mohamed I , Jenkins C , Godbole G . 2021. Phylogenomics and antimicrobial resistance of Salmonella Typhi and Paratyphi A, B and C in England, 2016-2019. Microb Genom 7:000633. doi:10.1099/mgen.0.000633 34370659 PMC8549371

[27] Kariuki S , Dyson ZA , Mbae C , Ngetich R , Kavai SM , Wairimu C , Anyona S , Gitau N , Onsare RS , Ongandi B , Duchene S , Ali M , Clemens JD , Holt KE , Dougan G . 2021. Multiple introductions of multidrug-resistant typhoid associated with acute infection and asymptomatic carriage, Kenya. Elife 10:e67852. doi:10.7554/eLife.67852 34515028 PMC8494480

[28] Gao Q , Liu Z , Xiang J , Zhang Y , Tong MX , Wang S , Zhang Y , Liu Q , Jiang B , Bi P . 2021. Impact of temperature and rainfall on typhoid/paratyphoid fever in Taizhou, China: effect estimation and vulnerable group identification. Am J Trop Med Hyg 106:532–542. doi:10.4269/ajtmh.20-1457 34872055 PMC8832923

[29] Saad NJ , Lynch VD , Antillón M , Yang C , Crump JA , Pitzer VE . 2018. Seasonal dynamics of typhoid and paratyphoid fever. Sci Rep 8:6870. doi:10.1038/s41598-018-25234-w 29720736 PMC5932015

[30] Phillips MT , Owers KA , Grenfell BT , Pitzer VE . 2020. Changes in historical typhoid transmission across 16 U.S. cities, 1889-1931: quantifying the impact of investments in water and sewer infrastructures. PLoS Negl Trop Dis 14:e0008048. doi:10.1371/journal.pntd.0008048 32187188 PMC7105137

[31] Dong B-Q , Yang J , Wang X-Y , Gong J , von Seidlein L , Wang M-L , Lin M , Liao H-Z , Ochiai RL , Xu Z-Y , Jodar L , Clemens JD . 2010. Trends and disease burden of enteric fever in Guangxi province, China, 1994-2004. Bull World Health Organ 88:689–696. doi:10.2471/BLT.09.069310 20865074 PMC2930361

[32] Zhang H , Zhang X , Yan M , Pang B , Kan B , Xu H , Huang X . 2011. Genotyping of Salmonella enterica serovar Typhi strains isolated from 1959 to 2006 in China and analysis of genetic diversity by genomic microarray. Croat Med J 52:688–693. doi:10.3325/cmj.2011.52.688 22180267 PMC3243322

[33] Feng Y , Zou S , Chen H , Yu Y , Ruan Z . 2021. BacWGSTdb 2.0: a one-stop repository for bacterial whole-genome sequence typing and source tracking. Nucleic Acids Res 49:D644–D650. doi:10.1093/nar/gkaa821 33010178 PMC7778894

[34] Ruan Z , Yu Y , Feng Y . 2020. The global dissemination of bacterial infections necessitates the study of reverse genomic epidemiology. Brief Bioinform 21:741–750. doi:10.1093/bib/bbz010 30715167

[35] Dyson ZA , Klemm EJ , Palmer S , Dougan G . 2019. Antibiotic resistance and typhoid. Clin Infect Dis 68:S165–S170. doi:10.1093/cid/ciy1111 30845331 PMC6405283

[36] Thanh Duy P , Thieu NTV , Nguyen Thi Nguyen T , Ngoc Dan Thanh H , Dongol S , Karkey A , Carey M , Basnyat B , Dougan G , Rabaa MA , Baker S . 2020. Gallbladder carriage generates genetic variation and genome degradation in Salmonella Typhi. PLoS Pathog 16:e1008998. doi:10.1371/journal.ppat.1008998 33085725 PMC7605710

[37] Prjibelski A , Antipov D , Meleshko D , Lapidus A , Korobeynikov A . 2020. Using SPAdes de novo assembler. Curr Protoc Bioinformatics 70:e102. doi:10.1002/cpbi.102 32559359

[38] Zhang S , den Bakker HC , Li S , Chen J , Dinsmore BA , Lane C , Lauer AC , Fields PI , Deng X . 2019. SeqSero2: rapid and improved Salmonella serotype determination using whole-genome sequencing data. Appl Environ Microbiol 85:e01746-19. doi:10.1128/AEM.01746-19 31540993 PMC6856333

[39] Yoshida CE , Kruczkiewicz P , Laing CR , Lingohr EJ , Gannon VPJ , Nash JHE , Taboada EN . 2016. The Salmonella in silico typing resource (SISTR): an open web-accessible tool for rapidly typing and subtyping draft Salmonella genome assemblies. PLoS One 11:e0147101. doi:10.1371/journal.pone.0147101 26800248 PMC4723315

[40] Chen J , Ed-Dra A , Zhou H , Wu B , Zhang Y , Yue M . 2022. Antimicrobial resistance and genomic investigation of non-typhoidal Salmonella isolated from outpatients in Shaoxing city, China. Front Public Health 10:988317. doi:10.3389/fpubh.2022.988317 36176509 PMC9513250

[41] Croucher NJ , Page AJ , Connor TR , Delaney AJ , Keane JA , Bentley SD , Parkhill J , Harris SR . 2015. Rapid phylogenetic analysis of large samples of recombinant bacterial whole genome sequences using Gubbins. Nucleic Acids Res 43:e15. doi:10.1093/nar/gku1196 25414349 PMC4330336

[42] Minh BQ , Schmidt HA , Chernomor O , Schrempf D , Woodhams MD , von Haeseler A , Lanfear R . 2020. IQ-TREE 2: new models and efficient methods for phylogenetic inference in the genomic era. Mol Biol Evol 37:2461. doi:10.1093/molbev/msaa131 32011700 PMC7182206

[43] Letunic I , Bork P . 2021. Interactive Tree Of Life (iTOL) v5: an online tool for phylogenetic tree display and annotation. Nucleic Acids Res 49:W293–W296. doi:10.1093/nar/gkab301 33885785 PMC8265157

[44] Cheng L , Connor TR , Sirén J , Aanensen DM , Corander J . 2013. Hierarchical and spatially explicit clustering of DNA sequences with BAPS software. Mol Biol Evol 30:1224–1228. doi:10.1093/molbev/mst028 23408797 PMC3670731

[45] Rambaut A , Lam TT , Max Carvalho L , Pybus OG . 2016. Exploring the temporal structure of heterochronous sequences using TempEst (formerly Path-O-Gen). Virus Evol 2:vew007. doi:10.1093/ve/vew007 27774300 PMC4989882

[46] Rieux A , Khatchikian CE . 2017. tipdatingbeast: an r package to assist the implementation of phylogenetic tip-dating tests using beast. Mol Ecol Resour 17:608–613. doi:10.1111/1755-0998.12603 27717245

[47] Duchêne S , Holt KE , Weill F-X , Le Hello S , Hawkey J , Edwards DJ , Fourment M , Holmes EC . 2016. Genome-scale rates of evolutionary change in bacteria. Microb Genom 2:e000094. doi:10.1099/mgen.0.000094 28348834 PMC5320706

[48] Bouckaert R , Vaughan TG , Barido-Sottani J , Duchêne S , Fourment M , Gavryushkina A , Heled J , Jones G , Kühnert D , De Maio N , Matschiner M , Mendes FK , Müller NF , Ogilvie HA , du Plessis L , Popinga A , Rambaut A , Rasmussen D , Siveroni I , Suchard MA , Wu C-H , Xie D , Zhang C , Stadler T , Drummond AJ , Pertea M . 2019. BEAST 2.5: an advanced software platform for Bayesian evolutionary analysis. PLoS Comput Biol 15:e1006650. doi:10.1371/journal.pcbi.1006650 30958812 PMC6472827

[49] Bortolaia V , Kaas RS , Ruppe E , Roberts MC , Schwarz S , Cattoir V , Philippon A , Allesoe RL , Rebelo AR , Florensa AF , Fagelhauer L , Chakraborty T , Neumann B , Werner G , Bender JK , Stingl K , Nguyen M , Coppens J , Xavier BB , Malhotra-Kumar S , Westh H , Pinholt M , Anjum MF , Duggett NA , Kempf I , Nykäsenoja S , Olkkola S , Wieczorek K , Amaro A , Clemente L , Mossong J , Losch S , Ragimbeau C , Lund O , Aarestrup FM . 2020. ResFinder 4.0 for predictions of phenotypes from genotypes. J Antimicrob Chemother 75:3491–3500. doi:10.1093/jac/dkaa345 32780112 PMC7662176

[50] Carattoli A , Hasman H . 2020. PlasmidFinder and in silico pMLST: identification and typing of plasmid replicons in whole-genome sequencing (WGS). Methods Mol Biol 2075:285–294. doi:10.1007/978-1-4939-9877-7_20 31584170

[51] Li Y , Teng L , Xu X , Li X , Peng X , Zhou X , Du J , Tang Y , Jiang Z , Wang Z , Jia C , Müller A , Kehrenberg C , Wang H , Wu B , Weill F-X , Yue M . 2022. A nontyphoidal Salmonella serovar domestication accompanying enhanced niche adaptation. EMBO Mol Med 14:e16366. doi:10.15252/emmm.202216366 36172999 PMC9641423

[52] Li Y , Ed-Dra A , Tang B , Kang X , Müller A , Kehrenberg C , Jia C , Pan H , Yang H , Yue M . 2022. Higher tolerance of predominant Salmonella serovars circulating in the antibiotic-free feed farms to environmental stresses. J Hazard Mater 438:129476. doi:10.1016/j.jhazmat.2022.129476 35809365

[53] Kang X , Zhou X , Tang Y , Jiang Z , Chen J , Mohsin M , Yue M . 2022. Characterization of two-component system CitB family in Salmonella pullorum. Int J Mol Sci 23:10201. doi:10.3390/ijms231710201 36077599 PMC9456408

[54] Chen J , Zhou X , Tang Y , Jiang Z , Kang X , Wang J , Yue M . 2023. Characterization of two-component system CitB family in Salmonella enterica serovar gallinarum biovar gallinarum. Vet Microbiol 278:109659. doi:10.1016/j.vetmic.2023.109659 36645991

[55] Achtman M , Zhou Z , Alikhan N-F , Tyne W , Parkhill J , Cormican M , Chiou C-S , Torpdahl M , Litrup E , Prendergast DM , Moore JE , Strain S , Kornschober C , Meinersmann R , Uesbeck A , Weill F-X , Coffey A , Andrews-Polymenis H , Curtiss Rd R , Fanning S . 2020. Genomic diversity of Salmonella enterica -the UoWUCC 10K genomes project. Wellcome Open Res 5:223. doi:10.12688/wellcomeopenres.16291.2 33614977 PMC7869069

