# Supplementary figures and images for: An integrated nationwide genomics study reveals transmission modes of typhoid fever in China
Source: mBio. 2023 Oct 6;14(5):e01333-23. doi: 10.1128/mbio.01333-23 (PMC10653838; doi:10.1128/mbio.01333-23)

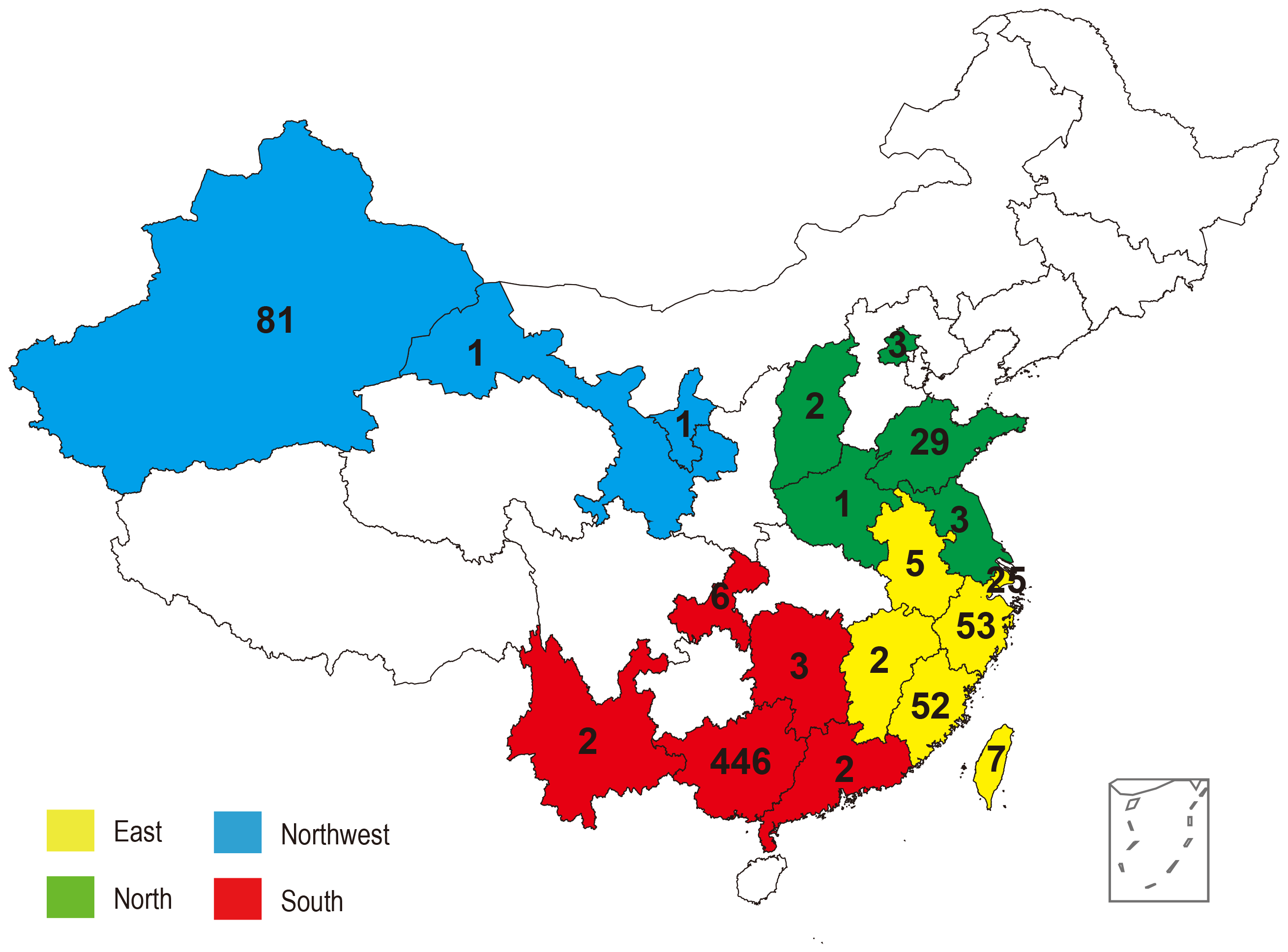

Supplement: Fig. S2 — Geographic analysis. [file mbio.01333-23-s0001.tif]

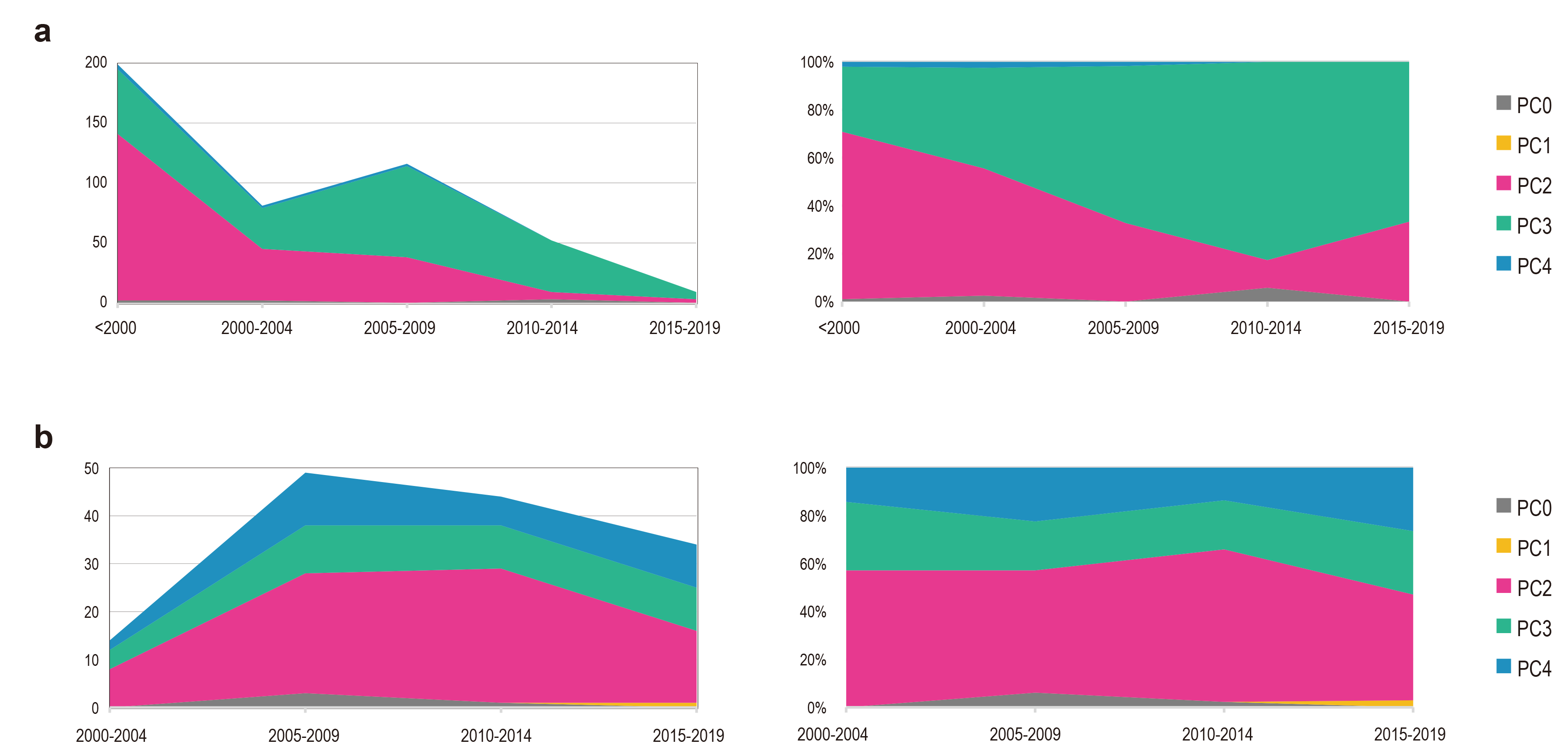

Supplement: Fig. S3 — Clonal replacement. [file mbio.01333-23-s0002.tif]

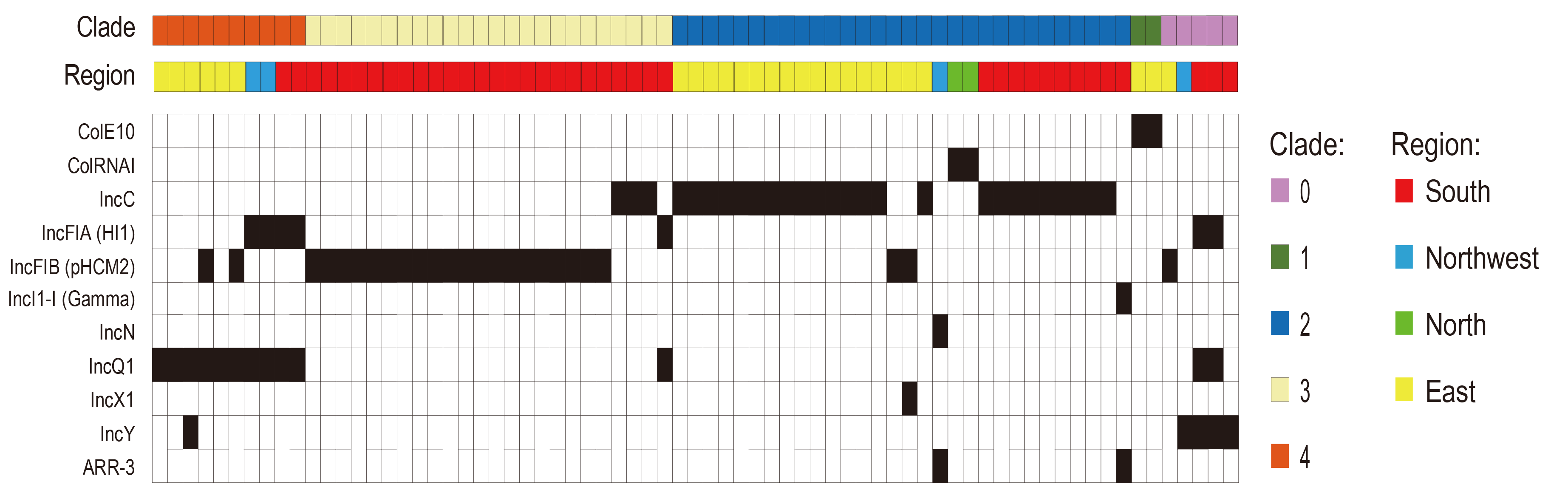

Supplement: Fig. S5 — Plasmid analysis. [file mbio.01333-23-s0004.tif]

## Root-to-tip regression

Clade 2.1

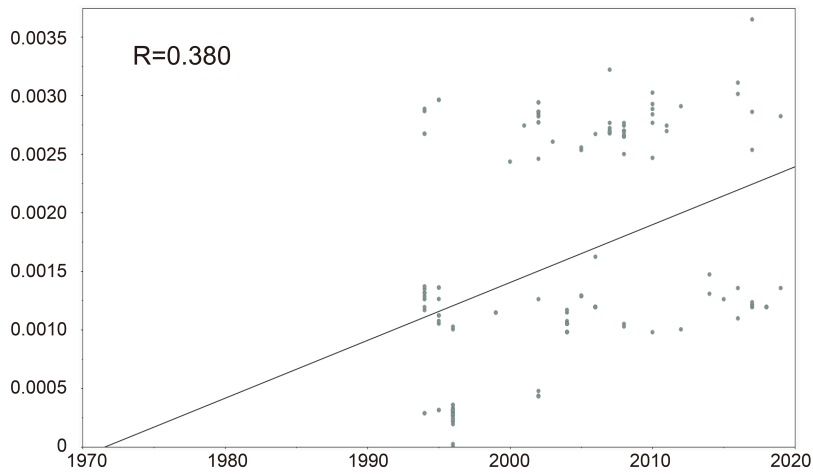

## Date-randomization test

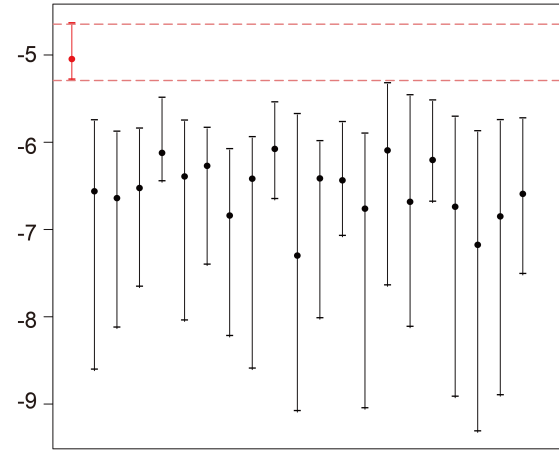

Clade 2.3

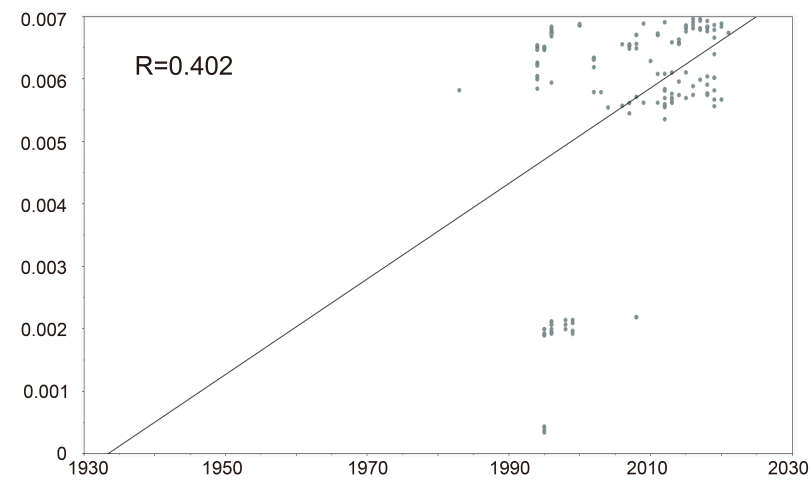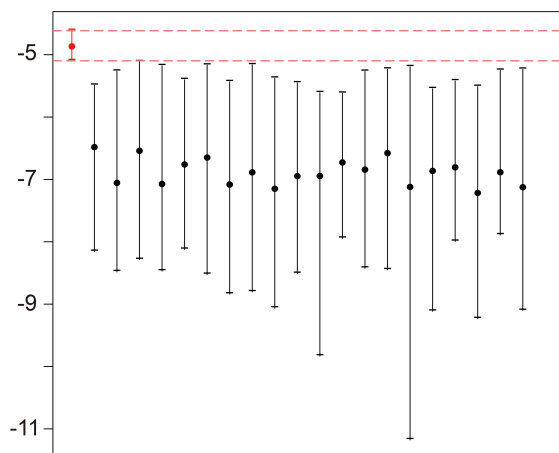

Clade 3.2

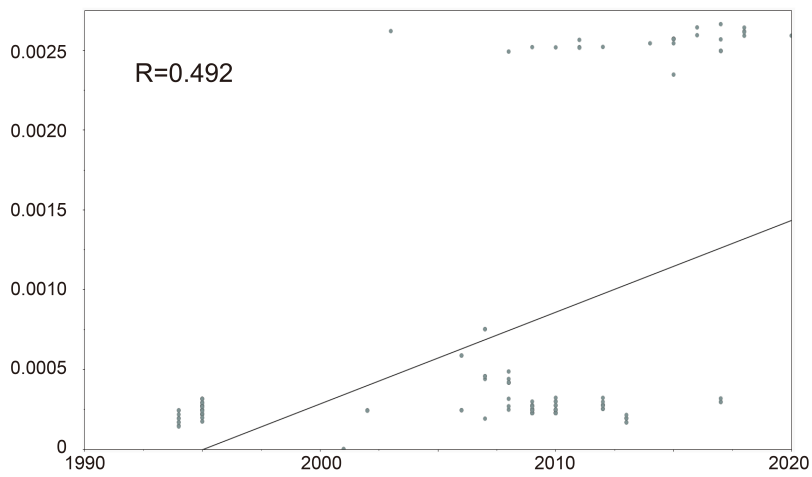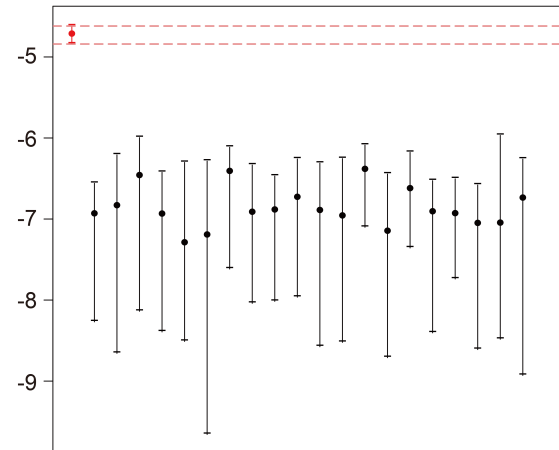

Clade 4.3

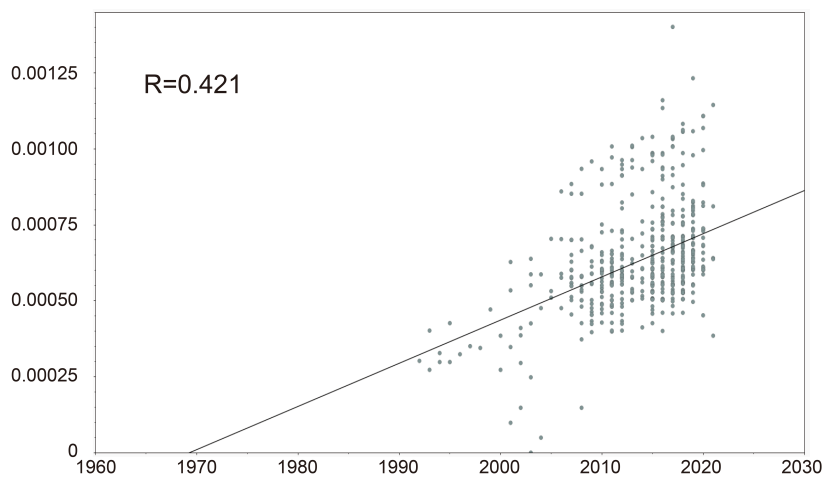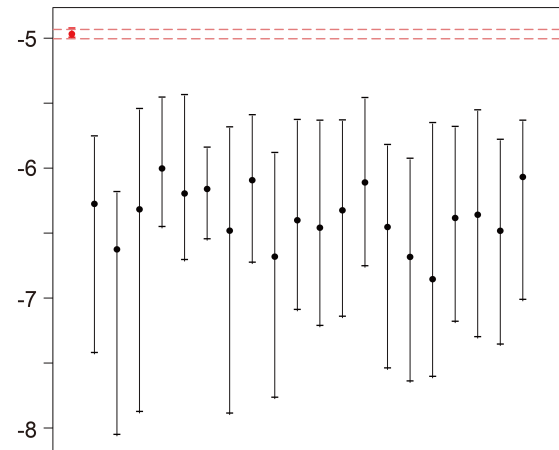

Supplement: Fig. S6 — Assessment of the temporal structure of clades. [file mbio.01333-23-s0005.pdf]

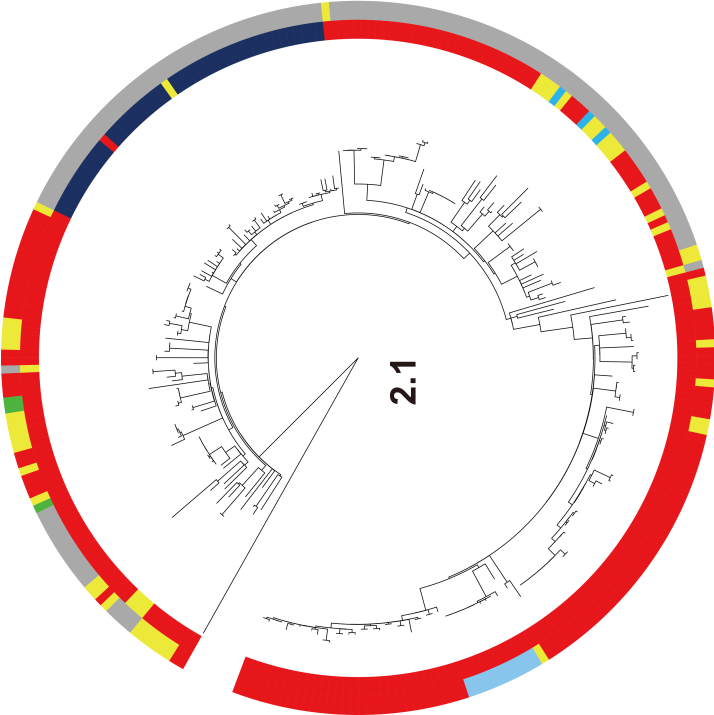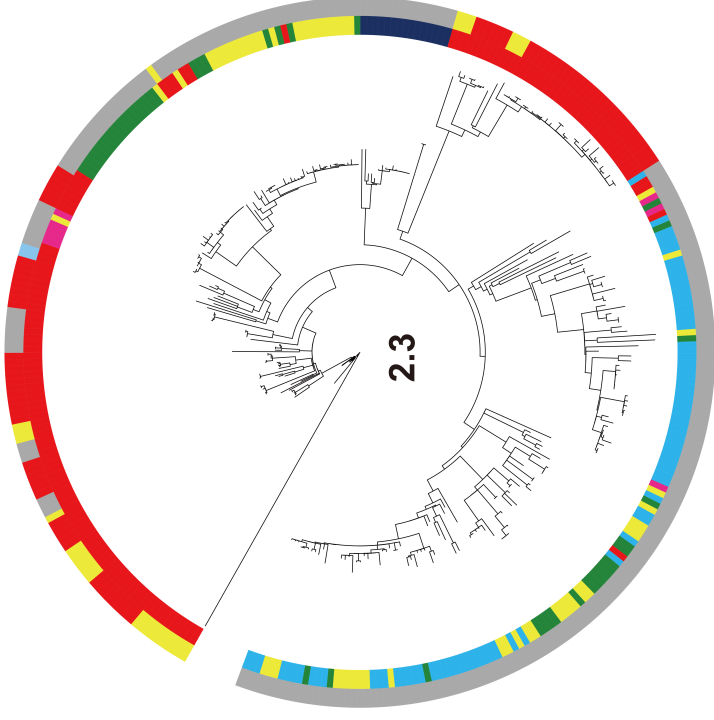

Outer ring (Regions in China):

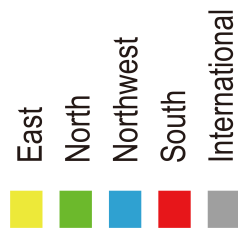

Inner ring (Continent):

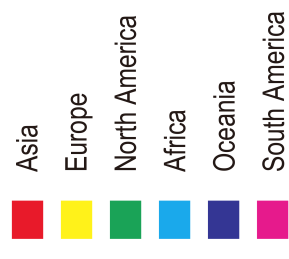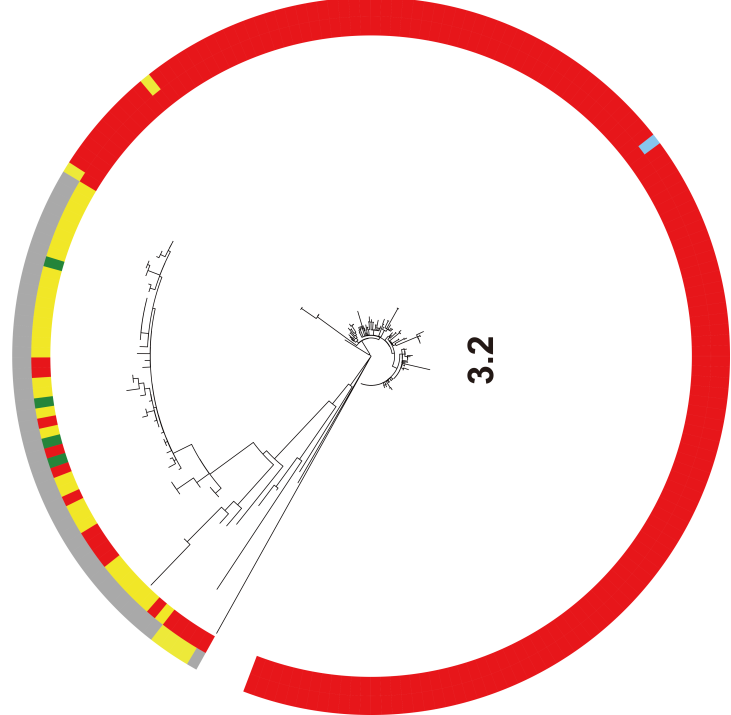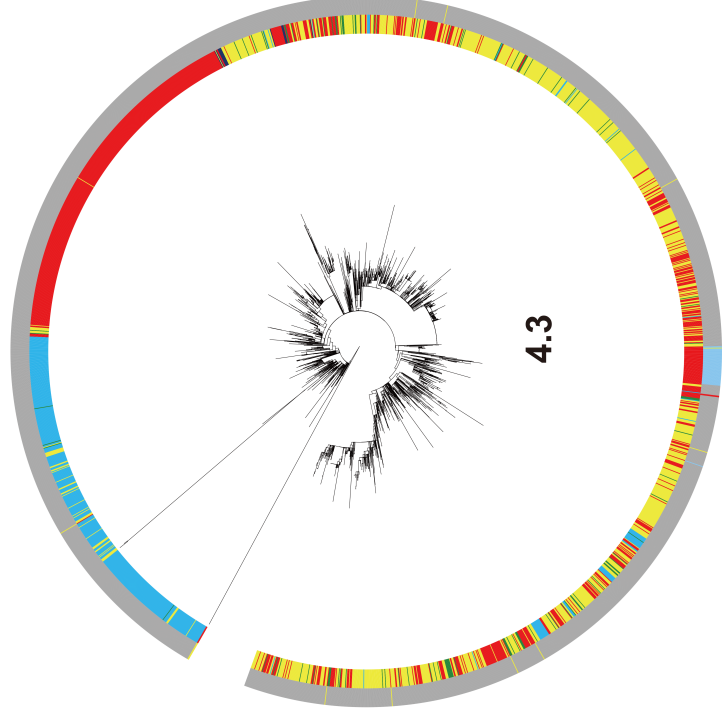

4.3

3.2

Supplement: Fig. S7 — ML tree. [file mbio.01333-23-s0006.pdf]

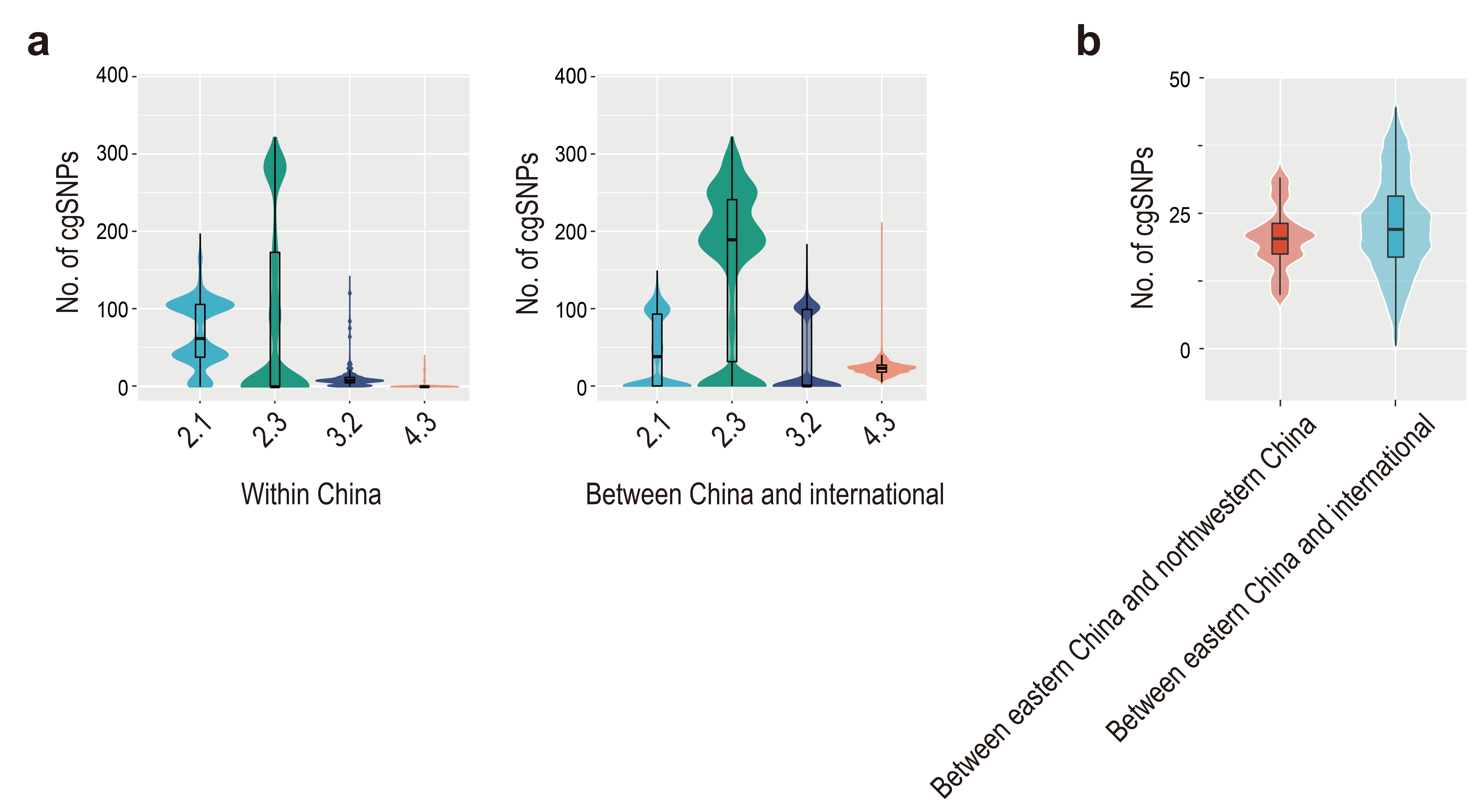

Supplement: Fig. S8 — Pairwise SNP. [file mbio.01333-23-s0007.tif]

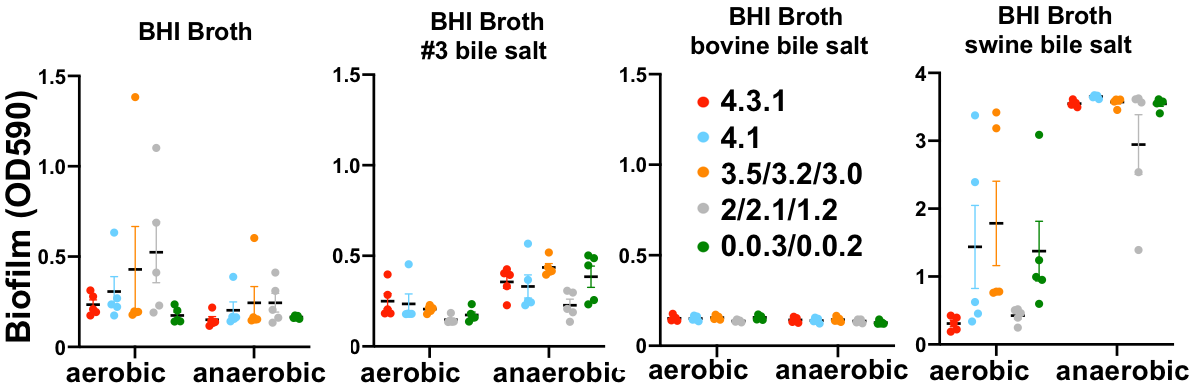

Supplement: Fig. S9 — Biofilm analysis. [file mbio.01333-23-s0008.tif]
