# Supplementary material for: An integrated nationwide genomics study reveals transmission modes of typhoid fever in China
Source: mBio. 2023 Oct 6;14(5):e01333-23. doi: 10.1128/mbio.01333-23 (PMC10653838; doi:10.1128/mbio.01333-23)
Supplement: Supplemental material — Supplemental legends and Fig. S1. [file mbio.01333-23-s0009.pdf]

Supplementary figures

Figure S1. Comparison of Genotyping Results Derived by BAPS and Genotyphi Software. The maximum-likelihood tree comprising the global collection of *S*. Typhi isolates is built based on cgSNP by IQ-Tree software. The global *S*. Typhi isolates are divided into five primary clades by the Genotyphi typing scheme. The tree branches are coloured according to the primary clades they belong to. The eight concentric rings from inner to outer: 1 and 2, primary clades and subclades designated by Genotyphi; 3 and 4, primary clades and subclades assigned by BAPS; 5, isolates from China (red) and other countries (grey).

Figure S2. The Number of Sequenced *S*. Typhi Isolates Collected from Different Provinces. These provinces are grouped into four regions according to climate and economic characteristics.

Figure S3. Temporal Variation of Lineage Composition of *S*. Typhi in China. (a) and (b) reflect the variation for isolates from southern and eastern China, respectively. The y-axis of the left plots reflects the absolute number of isolates, and the y-axis of the right plots reflects the percentage of each primary clade.

Figure S4. Age (a) and Sex (b) Composition of the Patients for Each Clade of *S*. Typhi in China in this study.

Figure S5. Plasmid Typing for *S*. Typhi in China. The plasmid replicon typing is performed by PlasmidFinder software. Each column indicates a Chinese *S*. Typhi isolate that carries a plasmid with known replicon types. In the grid, the black or white cell indicates the presence or absence of the plasmid.

Figure S6. Assessment of the Temporal Structure of the Clades 2.1, 2.3, 3.2 and 4.3. The left plots show the root-to-tip regression for the clades' maximum likelihood tree using TempEST software. Each point on the plot corresponds to a measurement from the root to each tip in the tree, and the solid line is the regression line. The right plots show the results of a date-randomization test using the TipDatingBeast R package. The y-axis shows the SNP evolutionary rate on a log10 scale, while the x-axis shows the actual date (red) as well as the twenty different replicates (black) of the date-randomization test. Points are mean estimates, and the lines are the Bayesian credible intervals around these values.

Figure S7. Maximum-likelihood Trees for Four *S*. Typhi Clades Dominant in China. The trees are built based on cgSNPs. The inner and outer rings indicate the continent and province (if collected from China) information where these isolates were collected.

Figure S8. The Within-clade Genetic Distance for the Four Dominant Clades in China. The genetic distance is represented by the number of pairwise cgSNP shown by the y-axis. (a) The left plot shows the distances for the isolates collected within China. The right plot shows the distance between the isolates from China and other countries. (b) The distances between the clade 4.3 from eastern China and the isolates from other regions. The distances between the 4.3 isolates from eastern China and the 4.3 isolates from other countries have a broader range, which starts from 0 cgSNP, indicating that the isolates from eastern China are much closer to specific international isolates than the isolates from northwestern China.

Figure S9. Biofilm formation in BHI and bile-associated medium under aerobic and anaerobic conditions. Experiments were performed using a microtiter plate reader, the y-axis scale shows the measurements at OD590 nm. Four groups of clinical isolates (Table S4) were chosen based on their lineage origin and availability in the

current collection. In general, all the isolates show strong biofilm formation capabilities under swine bile salt medium with anaerobic condition.

Supplementary tables

Table S1. Geographical distribution typhoidal incidence and their social-economic parameters.

Table S2. Summary of 731 *S*. Typhi Isolates from China.

Table S3. Summary of 5,164 *S*. Typhi isolates from countries around the world.

Table S4. *S*. Typhi isolates used for experiments in this study.

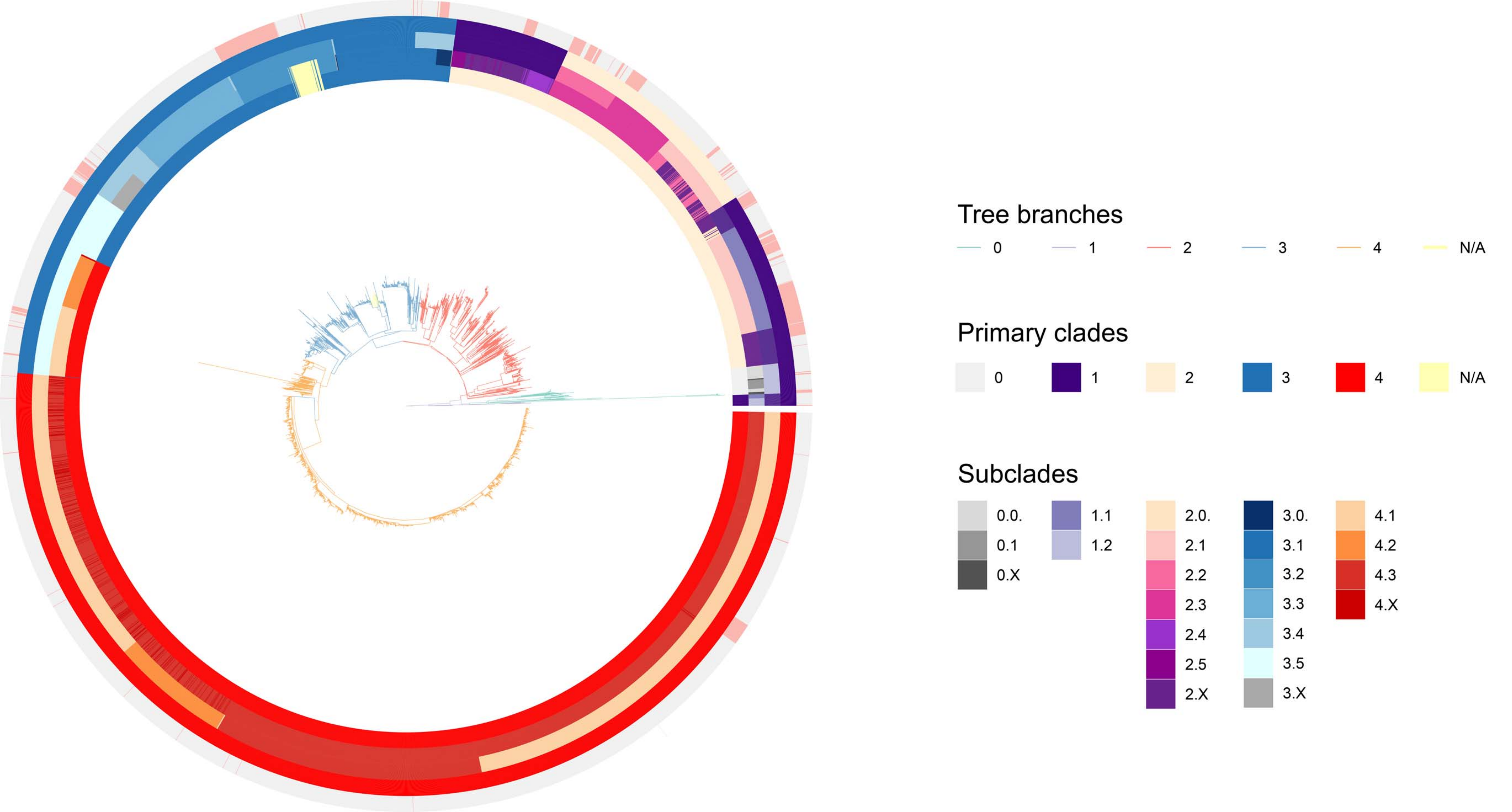

Tree branches
0
1
2
3
4
N/A
Primary clades
0
1
2
3
4
N/A
Subclades
0.0.
0.1
0.X
1.1
1.2
2.0.
2.1
2.2
2.3
2.4
2.5
2.X
3.0.
3.1
3.2
3.3
3.4
3.5
3.X
4.1
4.2
4.3
4.X
